# Genetic Admixture in the Culturally Unique Peranakan Chinese Population in Southeast Asia

**DOI:** 10.1093/molbev/msab187

**Published:** 2021-06-21

**Authors:** Degang Wu, Peter Yiqing Li, Bangfen Pan, Zenia Tiang, Jinzhuang Dou, Ivanna Williantarra, Amadeus Yeremia Pribowo, Rizky Nurdiansyah, The SG Peranakan Project, Roger S Y Foo, Chaolong Wang

**Affiliations:** 1 Department of Epidemiology and Biostatistics, Ministry of Education Key Laboratory of Environment and Health and State Key Laboratory of Environmental Health (Incubating), School of Public Health, Tongji Medical College, Huazhong University of Science and Technology, Wuhan, China; 2 Cardiovascular Research Institute, Yong Loo Lin School of Medicine, National University of Singapore, Singapore, Singapore; 3 NUHS Cardiovascular Diseases Translational Research Program, National University Health System, Singapore, Singapore; 4 Genome Institute of Singapore, Agency for Science, Technology and Research (A*STAR), Singapore, Singapore; 5 Department of Anatomy and Medical Imaging, School of Medical Science, Faculty of Medical and Health Sciences, The University of Auckland, Auckland, New Zealand; 6 Department of Biotechnology, Indonesia International Institute for Life Sciences (i3L), Jakarta, Indonesia; 7 Department of Bioinformatics, Indonesia International Institute for Life Sciences (i3L), Jakarta, Indonesia

**Keywords:** sex-biased admixture history, Chinese, Malays, whole-genome sequencing, mitochondrial haplogroups, Y haplogroups

## Abstract

The Peranakan Chinese are culturally unique descendants of immigrants from China who settled in the Malay Archipelago ∼300–500 years ago. Today, among large communities in Southeast Asia, the Peranakans have preserved Chinese traditions with strong influence from the local indigenous Malays. Yet, whether or to what extent genetic admixture co-occurred with the cultural mixture has been a topic of ongoing debate. We performed whole-genome sequencing (WGS) on 177 Singapore (SG) Peranakans and analyzed the data jointly with WGS data of Asian and European populations. We estimated that Peranakan Chinese inherited ∼5.62% (95% confidence interval [CI]: 4.76–6.49%) Malay ancestry, much higher than that in SG Chinese (1.08%, 0.65–1.51%), southern Chinese (0.86%, 0.50–1.23%), and northern Chinese (0.25%, 0.18–0.32%). A sex-biased admixture history, in which the Malay ancestry was contributed primarily by females, was supported by X chromosomal variants, and mitochondrial (MT) and Y haplogroups. Finally, we identified an ancient admixture event shared by Peranakan Chinese and SG Chinese ∼1,612 (95% CI: 1,345–1,923) years ago, coinciding with the settlement history of Han Chinese in southern China, apart from the recent admixture event with Malays unique to Peranakan Chinese ∼190 (159–213) years ago. These findings greatly advance our understanding of the dispersal history of Chinese and their interaction with indigenous populations in Southeast Asia.

## Introduction

The Straits of Malacca, which connects the Indian Ocean and the South China Sea trade networks, has become a global trading hub since the 15th century, epitomized by the establishment of the entrepot of Malacca (Freeman 2003). In the early days, foreign traders from all over the world met in entrepots along the Straits, conducting businesses while waiting for the favorable Monsoon season to embark on the next sea trip (Freeman 2003). Many foreigners established families and businesses in the Straits. Their descendants formed communities generally referred to by the locals as the Peranakans. The term “Peranakan” is an Indo-Malay word meaning “native-born.” Depending on their foreign ancestry, the Peranakans include Peranakan Chinese, Peranakan Indians, and Peranakan Eurasians, among whom, the Peranakan Chinese, also known as Straits-born Chinese, is the largest and most influential Peranakan community (Song 1984; Tan 2010; Lee 2013; Chia 2015). 

Although the first Chinese immigrants to Southeast Asia can be traced back to the tenth century, there was a major immigration wave of Chinese traders following the Seven Voyages of Zheng He in the 15th century, during which a treasure fleet of hundreds of ships led by the Chinese Admiral Zheng He expedited all the way from China to Southeast Asia, India, Persian Gulf, and East Africa (Heidhues 2013; Lockard 2013). With the reopening of Chinese–Malay trade relations, the Chinese migration wave to Southeast Asia persisted from the 15th to the 17th century, representing a major overseas dispersal in the Chinese population history. It is widely believed that the Peranakan Chinese are descendants of early Chinese traders from the southern provinces of China (Song 1984; Chia 2015). Through the years, the Peranakan Chinese community has become very influential in Southeast Asia and developed its unique culture, which preserves most of the Chinese traditions with strong Malay influences (Tan 2010). In particular, Baba Malay, a Malay-based creole spoken by Peranakans in Malacca, the Peranakan apparel, known as the sarong kebaya, and Peranakan food, a unique cuisine incorporating typical Malay spices, have become widely recognizable cultural symbols of the region.

In contrast to their famous hybrid culture, whether genetic admixture occurred alongside the cultural mixture remains debated and unverified, even within the Peranakan community (Chia 2015). Some maintain that they do not have Malay ancestry because by tradition, Peranakans married within their local Peranakan community or occasionally imported brides from China. The others believe intermarriage with local women must have occurred among their ancestors because the early Chinese immigrants were almost exclusively male traders, unaccompanied by their Chinese spouses (Tan 2010; Chia 2015). If there was genetic admixture, it is also unclear whether intermarriages occurred in the first few generations or was continuously persistent until the mass immigration of Chinese women began from the latter half of the 19th century (Tan 2010). Due to the scarcity of *bona fide* documents recording the ancestral lineage in the early days, there are no definitive answers to these questions despite strong public interests.

Among populations worldwide, it has often been observed that genetic admixture followed population dispersal, such as the Viking voyage of Scandinavian populations to the North Atlantic islands (Margaryan et al. 2020), the expansion of the Tibetan Empire to Central Asia (Yang et al. 2021), and the formation of African Americans and Latino populations in the Americas (Johnson et al. 2011; Moreno-Estrada et al. 2013; Bryc et al. 2015; Fortes-Lima et al. 2017). Cotransmission of genes and culture, such as languages, has been reported in admixed populations (Hunley et al. 2008; Verdu et al. 2017). Furthermore, the recent development of computational methods has enabled detailed inference of historical admixture events from present-day genomes, making population genomics invaluable in studying human population history, especially when historical records or archeological evidence are scarce (Alexander et al. 2009; Maples et al. 2013; Hellenthal et al. 2014; Fortes-Lima et al. 2017; Goldberg et al. 2017; Pierron et al. 2017). We have previously sequenced 4,810 Chinese, Malays, and Indians in the SG10K Pilot Project to characterize the broad Asian genetic diversity captured by Singapore (SG) populations, and found that Malays, who are representative of Southeast Asians, split from Chinese ∼24,800 years ago and experienced substantial admixture with Austronesians from East Asia ∼1,700 years ago (Wu et al. 2019). In the present study, we focus on reconstructing the admixture history of Peranakan Chinese by whole-genome sequencing (WGS) 177 Peranakans and joint analysis with data from the SG10K Pilot Project (Wu et al. 2019) and the 1000 Genomes Project (1KGP) (The 1000 Genomes Project Consortium 2015).

## Results

We sequenced 177 self-identified SG Peranakans at a mean depth of 15.2×. Demographic characteristics were shown in supplementary table S1, Supplementary Material online. Genotype calling and quality controls were performed jointly with samples from the SG10K Pilot Project (Wu et al. 2019). The final call set consisted of 90,008,560 SNPs and 9,300,998 insertions and deletions (INDELs) from autosomes and the X chromosome. After excluding close relatedness up to the third degree, 130 Peranakan samples were included in subsequent analyses.

### Population Structure

**Fig. 1.**
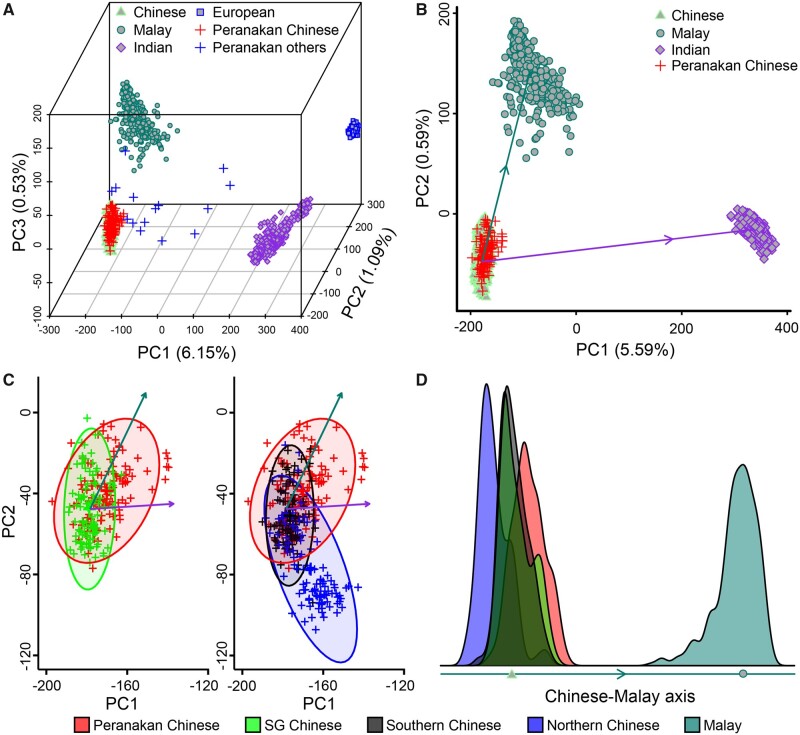
Population structure of Peranakan samples. (*A*) Projection of Peranakans onto the top 3 PCs of Chinese, Malays, Indians, and Europeans. We labeled Peranakans as Peranakan Chinese if they were within 3 standard deviations from the mean Peranakan coordinates in all 3 PCs. Numbers in the parentheses along axis labels are the proportions of variance explained by each PC. (*B*) Projection of Peranakan Chinese onto the top 2 PCs of Chinese, Malays, and Indians. The dark green and purple arrows connect the centroid of Chinese and the centroids of Malays and Indians, respectively. (*C*) Enlarged view of Peranakan Chinese, SG Chinese, southern Chinese, and northern Chinese projected onto the top 2 PCs of Chinese, Malays, and Indians. The shaded areas indicate the 95% concentration ellipses of each study population, and the meaning of dark green and purple arrows follow panel *B*. (*D*) Kernel density of samples from each population in panel *C* along the axis connecting the centroids of Chinese and Malays. Color key applies to both *C* and *D*.

We constructed a reference ancestry space by applying principal component analysis (PCA) on genotypes across 944,059 autosomal SNPs for 996 Chinese, 399 Malays, 629 Indians, selected from the SG10K Pilot Project (Wu et al. 2019), and 190 Europeans from 1KGP (The 1000 Genomes Project Consortium 2015) (Materials and Methods). These four reference populations, who are well separated by the top 3 principal components (PCs) (fig. 1*A*), represent local and immigrant populations in Singapore. We then projected Peranakans into the reference ancestry space (Wang et al. 2014, 2015). Peranakans largely overlapped with Chinese, except for a few outliers falling between the four reference populations (fig. 1*A*), consistent with their self-reported ancestry of Peranakan Indians, Eurasians, or Caucasians. In addition, some outliers might be introduced by very recent admixture events (i.e., within two generations), which may mask the admixture signals further back in time. Therefore, we excluded 15 Peranakan samples who were more than 3 standard deviations (SD) from the mean coordinates of Peranakans in any of the top 3 PCs, and focused on the remaining 115 samples, whom we labeled as Peranakan Chinese (Materials and Methods). PCA results based on 113,037 SNPs on the X chromosome resembled the autosomal PCA results for the reference populations (Procrustes similarity *t*_0_ = 0.91 for the top 3 PCs) (Wang et al. 2010), but had a noisier distribution for the Peranakans, indicating different admixture fractions on the X chromosome compared with the autosomes (supplementary fig. S2, Supplementary Material online).

After removing the outliers, we projected Peranakan Chinese, SG Chinese, and Han Chinese sampled from northern and southern China (CHB and CHS from 1KGP, respectively) onto the reference ancestry map spanned by the top 2 PCs of Chinese, Malays, and Indians (fig. 1*B*). Despite the substantial overlap between Peranakan Chinese and the reference Chinese, an enlarged view of the projected coordinates revealed a different distribution of Peranakan Chinese in comparison to SG Chinese, southern Chinese, and northern Chinese (fig. 1*C*). Specifically, we observed a trend of Peranakan Chinese tilted toward the reference Malays, indicating a low level of Malay admixture in Peranakan Chinese. Compared with the projected coordinates of southern Chinese, the mean shift toward Malays was insignificant for SG Chinese (*P *=* *0.31, *t*-test) but highly significant for Peranakan Chinese (*P *=* *7.5 × 10^−12^, fig. 1*D*).

We also examined the population structure among Peranakan Chinese and four reference populations using unsupervised ADMIXTURE analysis (supplementary fig. S3, Supplementary Material online) (Alexander et al. 2009; Alexander and Lange 2011). When the number of hypothetical ancestral components was set to *K *=* *4, the four components mostly align with the Chinese, Malay, Indian, and European ancestry. The fraction of the Malay-like component in Peranakan Chinese was significantly higher than that in the reference Chinese (10.1% vs. 6.78%, *P *=* *5.76 × 10^−9^, *t*-test). It is important to note that in unsupervised ADMXITURE analysis, shared ancestral components between populations could be attributed to demographic events other than genuine admixture (Lawson et al. 2018).

### Quantification of Admixture Levels

We calculated the *f*_3_ statistic (Patterson et al. 2012) to test potential admixture with Malays in Peranakan Chinese, SG Chinese, southern and northern Chinese (supplementary table S4, Supplementary Material online). Both Peranakan Chinese and SG Chinese had significantly negative *f*_3_ statistics (*Z* = −15.367 and −2.756, respectively), indicating admixture with Malays. In contrast, both southern and northern Chinese had positive *f*_3_ statistics, suggesting no admixture with Malays.

**Fig. 2.**
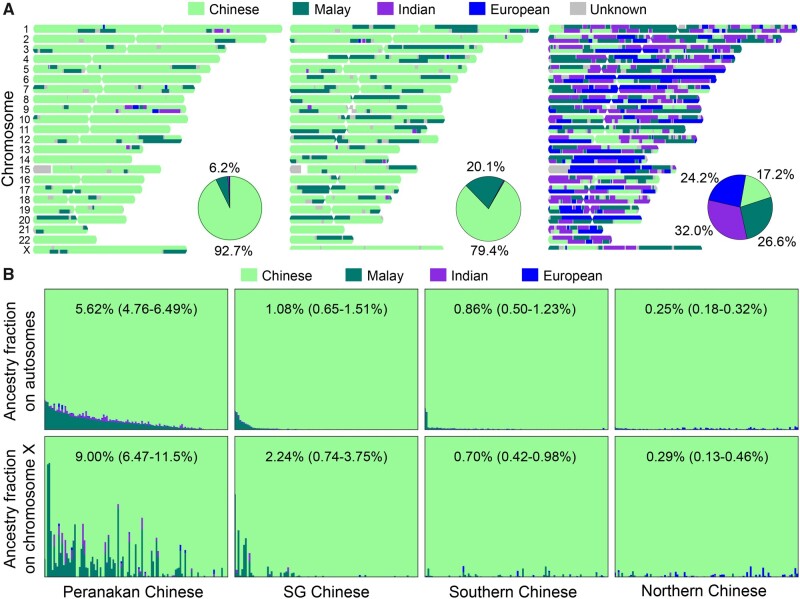
Admixture analysis using RFMix. (*A*) Local ancestry estimates of three Peranakans. From left to right: a female Peranakan Chinese with a typical fraction of Malay ancestry, a male Peranakan Chinese with a high fraction of Malay ancestry, and a male Peranakan Eurasian of complex multiway admixtures. Pie charts summarize the autosomal ancestry fractions of each individual. (*B*) Ancestry fractions on autosomes and the X chromosome for Peranakan Chinese, SG Chinese, southern Chinese, and northern Chinese. Each bar represents the ancestry composition of one individual. Individuals on the top and bottom panels are in the same order. The mean and 95% CI of Malay ancestry for each population are labeled within each panel.

To formally quantify the admixture proportions, we applied RFMix (Maples et al. 2013) to decompose individual genomes into local ancestry tracts, from which we estimated the global ancestry fractions (fig. 2; table 1). Figure 2*A* illustrates local ancestry tracts of three Peranakans: a typical Peranakan Chinese with ∼6.2% Malay ancestry, a Peranakan Chinese with ∼20.1% Malay ancestry, and a Peranakan Eurasian of complex multiway admixtures. The average Malay ancestry in Peranakan Chinese was 5.62% (95% CI: 4.76–6.49%) across autosomes, much higher than in SG Chinese (1.08%, 95% CI: 0.65–1.51%, *P *<* *2.2 × 10^−16^ by Welch’s *t*-test), southern Chinese (0.86%, 0.50–1.23%, *P *<* *2.2 × 10^−16^), and northern Chinese (0.25%, 0.18–0.32%, *P *<* *2.2 × 10^−16^, fig. 2*B*). The low levels of Malay ancestry estimated in southern and northern Chinese might reflect shared common ancestry rather than genuine admixture events. Similar trend was observed for ancestry fractions on the X chromosome, with mean Malay ancestry fraction in Peranakan Chinese, SG Chinese, southern Chinese, and northern Chinese being 9.00% (6.47–11.5%), 2.24% (0.74–3.75%, *P *=* *1.2 × 10^−5^ compared with Peranakan Chinese), 0.70% (0.42–0.98%, *P *=* *3.4 × 10^−9^) and 0.29% (0.13–0.46%, *P *=* *6.7 × 10^−10^), respectively (fig. 2*B*). For Peranakan Chinese, the average fraction of Malay ancestry on the X chromosome is higher than on the autosomes (9.00% vs. 5.62%, *P *=* *0.014 by paired Welch’s *t*-test), indicating sex-biased admixture history with higher contribution from Malay females than Malay males. The Malay fractions in autosomes and the X chromosome are moderately correlated (Spearman’s correlation *r*_s_ = 0.57, *P *=* *3.0 × 10^−11^, supplementary fig. S4*B*, Supplementary Material online) because of their different inheritance modes.

**Table 1.** Global Ancestry Fractions of Different Chinese Groups Inferred by RFMix.

Chinese group	Chr	Chinese %	Malay %	Indian %	European %
Peranakan	1-22	93.3 (92.3–94.2)	5.62 (4.76–6.49)	0.92 (0.73–1.11)	0.20 (0.13–0.27)
	X	89.7 (87.0–92.4)	9.00 (6.47–11.5)	1.13 (0.61–1.66)	0.16 (0.06–0.27)
Singapore	1-22	98.7 (98.3–99.2)	1.08 (0.65–1.51)	0.11 (0.07–0.15)	0.08 (0.06–0.09)
	X	97.6 (96.1–99.1)	2.24 (0.74–3.75)	0.14 (0.00–0.28)	0.02 (0.00–0.05)
Southern	1-22	98.9 (98.5–99.2)	0.86 (0.50–1.23)	0.06 (0.05–0.07)	0.20 (0.17–0.23)
	X	99.1 (98.8–99.4)	0.70 (0.42–0.98)	0.04 (0.01–0.07)	0.19 (0.06–0.31)
Northern	1-22	98.9 (98.8–99.0)	0.25 (0.18–0.32)	0.20 (0.17–0.23)	0.68 (0.58–0.77)
	X	98.8 (98.4–99.1)	0.29 (0.13–0.46)	0.15 (0.07–0.24)	0.79 (0.52–1.07)

Mean and 95% CI (in parentheses) of ancestry fractions are presented. “Chr” stands for chromosome.

We obtained similar results on the comparison of global admixture fractions between Peranakan Chinese and other Chinese groups using the supervised ADMIXTURE method (supplementary fig. S4*A*; table S3; Materials and Methods, Supplementary Material online). Nevertheless, admixture fractions derived from ADMIXTURE have a relatively weak correlation with those from RFMix for both autosomes (*r*_s_ = 0.40, *P *=* *7.5 × 10^−6^) and the X chromosome (*r*_s_ = 0.35, *P *=* *1.4 × 10^−4^, supplementary fig. S4*C*, Supplementary Material online), a phenomenon also noted by (Fortes-Lima et al. 2017) for low levels of admixture. In particular, ADMIXTURE tends to infer an excess of Malay ancestry, even in southern and northern Chinese sampled from China. It has been shown by simulation studies that the haplotype-based RFMix method outperforms the frequency-based ADMXITURE method in determining the ancestry fractions in complex admixed populations (Uren et al. 2020). Therefore, we chose to report the RFMix estimates as our main results and included the ADMIXTURE estimates in the supplements.

### Sex-Specific Contribution Inferred by MT and Y Haplogroups

**Fig. 3.**
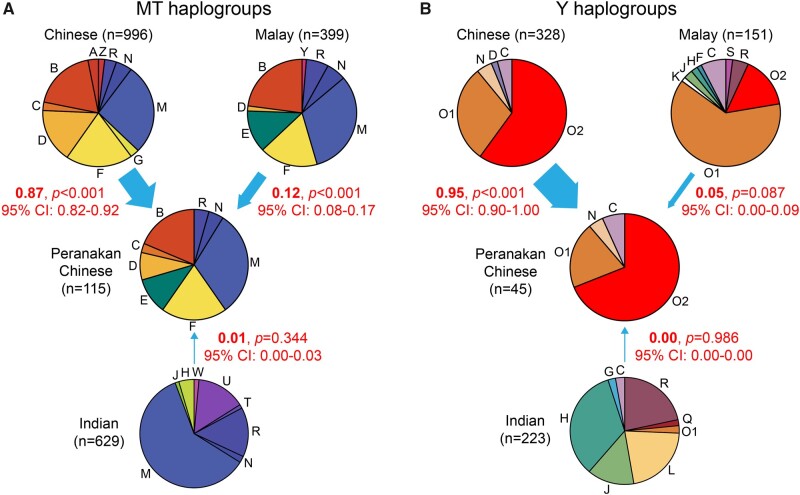
Maternal and paternal contribution to Peranakan Chinese from Chinese, Malays, and Indians. The estimates were based on MT haplogroup distributions (*A*) and Y haplogroup distributions (*B*), respectively, as indicated by red numbers along with the arrows in each panel. The 95% CI and *P* values were calculated by a simulation approach described in the Materials and Methods. The sample size for each population was indicated in the parentheses.

The availability of raw sequencing data for Peranakans and three SG populations (Chinese, Malays, and Indians) allowed us to directly assess sex-biased admixture by analyzing MT and Y haplogroups, the maternal and paternal lineage-specific markers, respectively (Materials and Methods). Consistent with population-genetic distances (Wu et al. 2019), Indians have distinct MT and Y haplogroup compositions from Peranakans, Chinese, and Malays (fig. 3; supplementary tables S5 and S6, Supplementary Material online). Despite similar MT haplogroup compositions in Chinese and Malays, haplogroup E, which was known to be geographically restricted to island southeast Asia (Soares et al. 2008; Ko et al. 2014), was found in 13% of Malays but only 0.5% in Chinese. In contrast, MT haplogroup D was rare in Malays (2%) but relatively common in Chinese (16%). Both MT haplogroups D and E were relatively common in Peranakan Chinese (8% and 10% for D and E, respectively), suggesting maternal contribution from both Chinese and Malays (fig. 3*A*). Combining evidence from all MT haplogroups, we estimated a significant proportion of maternal contribution from Malays (12%; 95% CI: 8–17%; *P *<* *0.001), whereas the majority were from Chinese (87%; 82–92%; *P *<* *0.001) and almost no contribution from Indians (1%; 0–3%; *P *=* *0.344).

We performed similar analysis on the Y haplogroups inferred from male samples. The O1 and O2 were the most ancestry-informative haplogroups given their dramatic frequency differences between Chinese and Malays, and near absence in Indians (fig. 3*B*). The ratio between O1 and O2 was 95/194 (=0.49) in Chinese, 93/23 (=4.04) in Malays, and 9/31 (=0.29) in Peranakan Chinese. The Y haplogroup composition in Peranakan Chinese was similar to Chinese (*P *=* *0.74 by Fisher’s exact test), but significantly different from Malays (*P *=* *3.0 × 10^−9^), suggesting almost exclusive paternal contribution from Chinese to Peranakan Chinese. We estimated 95% (90–100%; *P *<* *0.001) paternal contribution from Chinese, 5% (0–9%; *P *=* *0.087) from Malays, and 0% (0–0%; *P *=* *0.986) from Indians. The paternal contribution from Malays was not significantly greater than 0.

### Inference of Admixture History

**Fig. 4.**
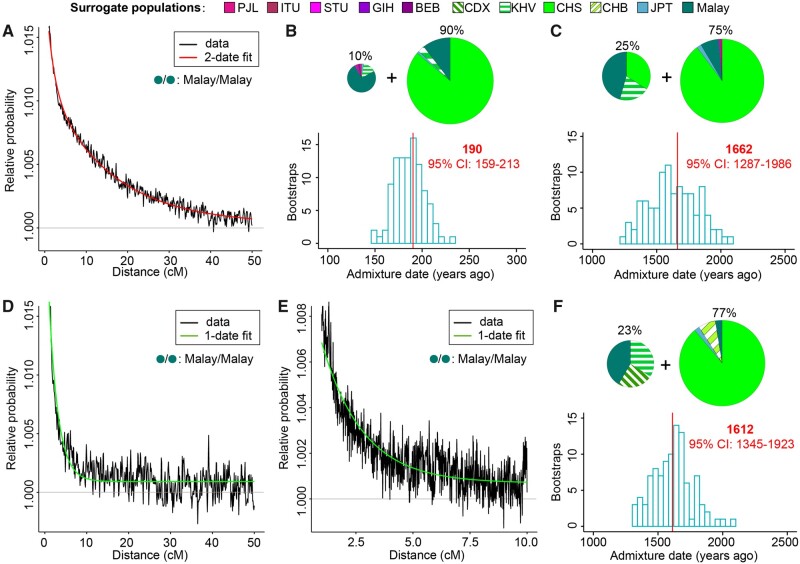
Inference of admixture date and source composition using GLOBETROTTER for Peranakan Chinese (*A–C*) and SG Chinese (*D–F*). The coancestry curves display the relative probability that two genomic chunks at a certain genetic distance were copied from a pair of surrogate populations. GLOETROTTER jointly fits all coancestry curves by either a one-date or a two-date admixture model. (*A*) The Malay–Malay coancestry curve of Peranakan Chinese, fitted by a two-date model. (*B*, *C*) Source compositions (upper) and date (lower) of the recent admixture event (*B*) and the ancient admixture event (*C*). The percentages above each pie chart represent the contribution from each source population, whose genetic background can be approximated by the surrogate populations shown in the pie chart. The histogram indicates the distribution of the estimated admixture dates based on 100 bootstraps, whereas the red vertical line indicates the point estimate using the original data. (*D*, *E*) The Malay/Malay coancestry curve of SG Chinese at genomic range of 0–50 cM (*D*) and 0–10 cM (*E*), fitted by a one-date model. The finer scale of 0–10 cM provides a higher resolution to infer the ancient admixture event. (*F*) Source compositions (upper) and date (lower) of the inferred admixture event. Abbreviations of 1KGP populations: BEB, Bengali; GIH, Gujarati; ITU, Telugu; PJL, Punjabi; STU, Sri Lankan Tamil; CDX, Chinese Dai; CHB, Han Chinese in Beijing; CHS, Southern Han Chinese; JPT, Japanese; KHV, Kinh in Vietnam.

Finally, we performed demographic inference of admixture events using GLOBETROTTER (Hellenthal et al. 2014). We included East Asian and South Asian populations from 1KGP and Malays as the surrogate populations of ancestral sources. The geographic distribution of the populations was shown in supplementary figure S5, Supplementary Material online. In particular, the 1KGP East Asian populations include Kinh from Vietnam (KHV) and Dai from southwestern China (CDX), both having close genetic relationship to Austronesian people who expanded across Southeast Asia from Taiwan (McColl et al. 2018). For comparison, we analyzed Peranakan Chinese and SG Chinese independently as the target population. Coancestry curves at a grid range of 1–50 cM for pairs of surrogate populations are displayed in supplementary figures S6 and S7, Supplementary Material online. In particular, the Malay–Malay coancestry curve, which shows the probability that two genomic segments at a given distance are both from the Malay population, decays exponentially for both Peranakan Chinese and SG Chinese, leading to the rejection of the null hypothesis of no admixture for both groups (empirical *P *<* *0.01; figs. 4*A* and *D*). The decaying speed for Peranakan Chinese, however, is slower than that for SG Chinese, indicative of different admixture histories.

GLOBETROTTER determined that the admixture history of Peranakan Chinese could be best described by a multiple-date-two-way admixture model, which involves more than one pulse of admixture between two source populations. By fitting a two-date-two-way admixture model for Peranakan Chinese and assuming 29 years per generation (Hellenthal et al. 2014), we estimated a recent admixture event happened ∼190 (95% CI: 159–213) years ago with 10% contribution from a Malay-dominant source and 90% contribution from a CHS-dominant source (fig. 4*B*), and an ancient admixture event happened ∼1,662 (1,287–1,986) years ago with 25% contribution from a source mixed by Malays, KHV, and CHS, and 75% contribution from a CHS-dominant source (fig. 4*C*). The estimated 10% contribution from a Malay-dominant source in the recent admixture event was generally consistent with (although slightly higher than) the Malay contribution derived from RFMix and haplogroup analyses (figs. 2 and 3).

In contrast, the best-guess model of SG Chinese was a one-date-two-way admixture model. Considering the rapid decay of coancestry curves, we reran GLOBETROTTER to fit the coancestry curves at a finer grid range of 1 to 10 cM for more accurate admixture dating (fig. 4*E* and supplementary fig. S8, Supplementary Material online). We estimated an ancient admixture event ∼1,612 (95% CI: 1,345–1,923) years ago with 23% contribution from a source mixed by Malays, KHV, and CDX and 77% contribution from a CHS-dominated source (fig. 4*F*). Both the admixture date and ancestral source compositions were highly similar to the ancient admixture event in the Peranakan Chinese (fig. 4*C*), consistent with a shared admixture event that occurred in the common ancestors of Peranakan Chinese and SG Chinese.

## Discussion

Coupling large-scale WGS data with state-of-the-art computational methods, we have performed comprehensive genomics analysis to characterize the admixture history of Peranakan Chinese in Singapore. Compared with microarray genotyping data, WGS data are free of potential Eurocentric ascertainment bias (Patterson et al. 2012), which is particularly important for genetic studies of Asian populations. We have detected a significant Malay ancestry component in Peranakan genomes, ranging from 5% to 10% based on different methods and genetic markers, much higher than those in the general SG Chinese, southern Chinese, and northern Chinese. This finding strongly supports the hypothesis that genetic admixture co-occurred with the cultural mixture in the formation of the Peranakan Chinese community (Song 1984; Tan 2010; Lee 2013; Chia 2015). In comparison, Indian and European ancestry components in Peranakan Chinese were negligible.

The complete genetic fingerprints obtained by WGS of both study and reference populations, including sex-linked markers on the X and Y chromosomes and the MT DNA, enabled us to study potential sex bias among the Chinese and Malay ancestors of Peranakan Chinese (Song 1984; Tan 2010; Lee 2013; Chia 2015). Consistent with the observation on the X chromosomes, we estimated based on the uniparental MT and Y haplogroups that the Malay ancestry in Peranakan Chinese was primarily contributed by Malay females rather than males. If we assumed equal numbers of males and females among the founders of Peranakan Chinese, the female to male ratio is approximately 0.92 (=87%/95%) from Chinese and 2.4 (=12%/5%) from Malays, despite insignificant contribution from Malay males (5%, *P *=* *0.087, fig. 3). These results are consistent with the hypothesis that early Chinese traders wedded local Malay females, due to the lack of Chinese females among the early immigrants (Song 1984; Tan 2010; Lee 2013; Chia 2015). Besides, native Malay wives could have helped early Chinese traders with their businesses in local communities (Tan 2010). Some Malay females may have been adopted into the Peranakan Chinese community at a young age (Lee 2013). Furthermore, due to the patrilineality traditions of both the Malay and Chinese communities, offspring of intermarriages between female Peranakan Chinese and male Malays would be absorbed into the Malay community instead of the Peranakan Chinese community (Tan 2010). It is possible but likely very rare that Malay males contributed to Peranakan Chinese community by adoption.

The distribution of ancestral haplotype tracts across the genome suggested multiple waves of admixture in the ancestors of Peranakan Chinese. By fitting a two-date-two-way model, we estimated a recent admixture event dated ∼190 (95% CI: 159–213) years ago, prior to the massive immigration of Chinese to Singapore starting from 1850s (Lim 2013), and an ancient admixture event dated ∼1,662 (1,287–1,986) years ago. The recent admixture event was unique to Peranakan Chinese, reflecting the intermarriage between local Malays and the early Chinese traders after settling in the Malay Archipelago between the 15th and the 17th centuries (Song 1984; Tan 2010; Lee 2013; Chia 2015). The inferred admixture date of ∼190 years ago, however, is more recent than the settlement date of the early Chinese traders proposed by historians. The inconsistency may reflect a continuous or multiple-wave admixture history between the Peranakan community and the Malays, for which the GLOBETROTTER method tends to infer the latest date of admixture (Hellenthal et al. 2014). Interestingly, the inferred admixture date coincided with the founding of Singapore as a British trading post in 1819, immediately after which many Peranakans reportedly rushed to Singapore from Malacca, Penang, and Batavia for business opportunities (Song 1984). It is also possible that Peranakan Chinese in Singapore experienced more recent admixture than those in other straits settlements, as observed in 1914 that the majority of Singapore Peranakan Chinese was “of the 3rd and 4th in descent from a purely Chinese male progenitor,” whereas the Peranakan Chinese in other straits settlements could be “5th or 6th” descendants from the pure Chinese male ancestor (Song 1984).

An ancient admixture event was detected in both Peranakan Chinese and SG Chinese with similar admixture source compositions and dates. Because most SG Chinese are descendants of southern Chinese (Wu et al. 2019), this admixture event is likely related to the formation of modern southern Han Chinese. The admixture date inferred from SG Chinese was ∼1,612 (1,345–1,923) years ago, corresponding to the period from Eastern Han (25–220 CE) to Tang Dynasty (618–907 CE) in China. This was a volatile period characterized by frequent wars and political instability in central China (Schottenhammer 2013). Massive Han Chinese migrated from central China to the south due to historical civil wars, such as the An Lushan Rebellion (755–763 CE) and the Huang Chao Rebellion (875–884 CE) (Schottenhammer 2013). Notably, the central government of Tang Dynasty established the prefecture of Zhangzhou in 686 CE and sinicized various local tribes in southern China, who were closely related to the Austronesian peoples in Taiwan (Sanchez-Mazas et al. 2008; Ko et al. 2014). Here we use the term “early Austronesian” to represent local indigenous people in coastal mainland southern China before the southward expansion of Han Chinese. Ancient admixture likely occurred between Han Chinese migrated from central China with the local tribes in southern China, forming the present-day southern Han Chinese. This hypothesis is consistent with inferred admixture sources where 23% contribution comes from a source whose haplotypes can be found in the present-day Malays, KHV, and CDX (fig. 4*F*), all closely related to Austronesians.

**Fig. 5.**
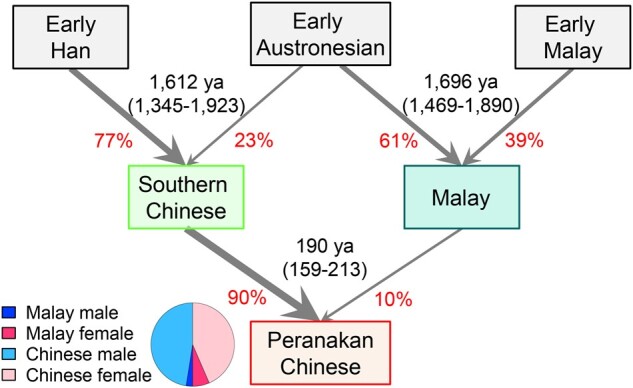
Diagram of the admixture history of Peranakan Chinese. Admixture dates (95% CI in parentheses) and relative contributions from each ancestral source (in red) were obtained from GLOBETROTTER analysis. The admixture event in Malays dated ∼1,696 years ago (ya) was described in our previous study (Wu et al. 2019). The piechart represents sex-specific contributions, based on MT and Y haplogroups, from Chinese and Malays to the Peranakan Chinese community, assuming equal numbers of males and females in the founders of Peranakan Chinese.

Austronesian peoples are well-known for their maritime expansion, originating from Taiwan ∼4,000 years ago and gradually colonizing a large part of the Indo-Pacific region (Sanchez-Mazas et al. 2008; Lipson et al. 2014; McColl et al. 2018). We have previously inferred the present-day Malays as descendants from the admixture between Austronesians (61%) and early Malays (39%) dated 1,696 (95% CI: 1,469–1,890) years ago (Wu et al. 2019). We did not detect this admixture event using the Peranakan Chinese genomes, likely because the signal was too weak given the relatively small genetic contribution from Malays to Peranakan Chinese. Taking together, we propose a simplistic admixture history of Peranakan Chinese, whose two ancestral populations, southern Chinese and Malays, were populations descending from early Han and early Malays who also experienced admixture with early Austronesians, respectively (fig. 5).

To summarize, we have reconstructed the genetic admixture history of Peranakan Chinese using WGS data from both our target population and relevant reference populations, which are only recently available with the advances in human population genomics (The 1000 Genomes Project Consortium 2015; Wu et al. 2019). A few limitations should be noted. First, the Peranakan communities across Southeast Asia are very diverse, including Peranakan Indians, Peranakan Eurasians, and Peranakan Chinese at different settlement sites, and each may have gone through a different history in interacting with local people (Tan 2010; Lee 2013). The present study was limited to the Peranakan Chinese due to the small number of other Peranakans in our recruited samples. Second, because Malays are genetically similar to Chinese with *F*_ST_ = 0.012 (Wu et al. 2019), it would be difficult to accurately distinguish ancestral tracts of Chinese and Malay origins, leading to uncertainty in the estimated admixture fractions. Third, the GLOBETROTTER method, which we used to infer the date and source of admixture events, has difficulty modeling complex admixture history with more than two dates or multiple sources (Hellenthal et al. 2014). Thus, we used simple one-date-two-way or two-date-two-way models for approximation of the major admixture events. Despite the limitations, evidence based on a variety of computational methods and genetic markers consistently suggested a moderate but significant level of Malay admixture in Peranakan Chinese, primarily contributed by Malay females about two centuries ago. Furthermore, we found evidence of admixture with early Austronesian-related peoples in the formation of modern southern Han Chinese. These genetic findings greatly enhance our understanding of the origin of Peranakan Chinese and the historical interactions between Chinese and indigenous populations in Southeast Asia.

## Materials and Methods

### Sample Collection

We recruited self-identified Singapore Peranakan participants by distributing research advertisements through the Peranakan Association Singapore, the Gunong Sayang Association, the Peranakan Magazine, and social media. Blood samples from 177 participants were collected following an informed consent protocol and procedure approved by the Institutional Review Board of National University of Singapore (Approval: H-17-049). These samples were sequenced as part of the SG10K Project and 79 out of 177 samples have previously been included in the SG10K Pilot study (Wu et al. 2019).

### DNA Extraction, Library Preparation, and Sequencing

The experimental procedure was as described for the SG10K Pilot study (Wu et al. 2019). Briefly, genomic DNA was extracted using PureLink (ThemoFisher) and quantified by Qubit dsDNA HS Assay Kit (Life Technologies). DNA integrity was interrogated using Qubit dsDNA HS Standard #2 DNA and 1 kb DNA ladder (New England Biolabs) on 1% GelRed (Biotium) stained Hyagarose (Hydrogene) agarose gel at 100–120 volts for 60 minutes. Library preparation was undertaken as per protocol using NEBNext Ultra II DNA Library Prep Kit for Illumina (New England Biolabs). Paired-end 151 bp WGS with an insert size of 350 bp was performed using Illumina X platform with HiSeq X Ten Reagent Kit v2.5 (Illumina). The target sequencing depth was 15× for all samples.

### Genotype Calling and Phasing

We aligned sequencing reads to the human reference genome GRCh37 with decoy sequences (hs37d5) using BWA-MEM (v 0.7.12; -M) (Li 2013), removed PCR duplicates with samblaster (v 0.1.22) (Faust and Hall 2014), and performed base quality recalibration using BamUtil recab (v 1.0.13; --maxBaseQual 40). We used VerifyBamID (v 1.1.2; --precise --maxDepth 100 --minMapQ 20 --minQ 20 --maxQ 100) to estimate sequencing depth and DNA contamination rate based on 1,285,226 autosomal SNPs with MAF > 0.05 overlapped with the SGVP data set (Teo et al. 2009; Jun et al. 2012). All samples had estimated contamination rate <0.002 and the average sequencing depth is 15.2×.

Following the pipeline of the SG10K Pilot Project (Wu et al. 2019), we performed joint genotype calling, quality controls, and haplotype phasing of 177 Peranakan samples and 4,731 samples from other cohorts in the SG10K Pilot Project (Wu et al. 2019). Importantly, we employed LD-based joint calling and phasing of all 4,908 samples together to ensure high-quality haplotypes (Browning and Yu 2009; Loh et al. 2016). The final call set consists of 86,678,694 SNPs and 8,947,718 insertions and deletions (INDELs) on autosomes, and 3,329,866 SNPs and 353,280 INDELs on the X chromosome.

### Inference of Genetic Relatedness

We used PC-Relate (v 2.2.2) (Conomos et al. 2016) to estimate kinship coefficient φ and the probability of zero identity-by-descent sharing (π_0_) between each pair of individuals, who would be classified as *k*-degree related if their φ was within the range of (2^-k-1.5^, 2^-k-0.5^) (Manichaikul et al. 2010). We inferred 96 closely related pairs (up to third degree) among Peranakans (supplementary table S2, Supplementary Material online), consistent with their self-reported relatedness. By excluding 47 samples involved in multiple related pairs, we obtained an unrelated set of 130 Peranakans.

### Reference Populations

Given the immigrant history of Singapore, we constructed an ancestry reference panel consisting of Chinese, Malays, Indians, and Europeans. We combined CEU and GBR samples from 1KGP to form the European group (*n* = 190) (The 1000 Genomes Project Consortium 2015). We selected Chinese, Malays, and Indians from the Singapore Epidemiology of Eye Diseases cohort (SEED, *n* = 1,536) and the Tan Tock Seng Hospital cohort (TTSH, *n* = 971), two largest cohorts in the SG10K Pilot Project (Wu et al. 2019). We took the following steps to exclude potential recent admixed samples. First, we performed PCA on the genotyping data of the Singapore Genome Variation Project (SGVP), consisting of 96 Chinese, 89 Malays, and 83 Indians, whose four grandparents were confirmed to have the same ethnicity (Teo et al. 2009). PCA was performed on 1,285,226 autosomal SNPs with MAF > 0.01 using LASER (v 2.04, options: trace -K 20 -k 3) (Wang et al. 2014). Second, we calculated the 95% concentration ellipses of the three populations by assuming the first 2 PCs of subjects from each population followed a bivariate Gaussian distribution (supplementary fig. S1*A*, Supplementary Material online). Third, we projected SEED and TTSH samples onto the SGVP map (Wang et al. 2015) and excluded samples outside of the 95% concentration ellipses. Among the remaining samples, we further excluded samples with inferred genetic sex or ethnicity different from self-reported information, sequencing depth <8×, or close relatedness. The final reference panel consisted of 996 (male/female, 328/668) Chinese, 399 (151/248) Malays, and 629 (223/406) Indians.

### Chinese Populations for Comparison

In parallel to the analysis of Peranakan Chinese, we analyzed three other Chinese groups for comparison. The SG Chinese group included 100 Chinese (male/female, 50/50) randomly selected from the Heart Failure cohort of the SG10K Pilot Project (Wu et al. 2019), excluding potential recent admixed samples using the same procedure described in the “Reference populations” section. These samples represent the general SG Chinese and possibly include some Peranakan Chinese. We also included 103 Han Chinese from Beijing (CHB) and 105 Han Chinese from south China (CHS), both from 1KGP (The 1000 Genomes Project Consortium 2015), to represent northern and southern Chinese, respectively.

### SNP QC

We merged our call set with the 1KGP Phase 3 data set (The 1000 Genomes Project Consortium 2015), by taking the intersection and excluding multiallelic SNPs, INDELs, SNPs within 5 bp of any INDELs, SNPs with mismatched alternative alleles in two data sets, and the pseudo-autosomal regions (PARs) from chromosome X, resulting in 26,743,581 autosomal SNPs and 945,337 SNPs on the X chromosome. We then extracted Peranakan samples and the selected reference populations, and removed SNPs with MAF < 0.05, resulting in 5,336,958 autosomal SNPs and 155,049 SNPs on chromosome X. We thinned the autosomal SNPs to be at least 2 kb apart from each other resulting in 983,282 SNPs (Chang et al. 2015). For PCA and ADMIXTURE (Alexander et al. 2009) analysis, we further excluded 27 long-range LD regions on autosomes, which might introduce artifact patterns (Price et al. 2008; Wu et al. 2019), resulting in 944,059 autosomal SNPs. For the X chromosome, we coded genotypes for males as homozygotes and applied PCA on the combined data set of Peranakans and four reference populations, and iteratively removed outlier SNPs that had PC loadings >5 standard deviations (SD) away from the average loadings among the top 10 PCs, resulting in 113,037 SNPs.

### PCA and Identification of Outlier Samples

We performed PCA on the four reference populations using 944,059 autosomal SNPs and 113,037 chromosome X SNPs, respectively (Wang et al. 2014). We then projected Peranakans, SG Chinese, CHB, and CHS samples into the reference ancestry spaces of top 3 PCs using LASER (options: trace -K 20 -k 3) (Wang et al. 2015). To separate Peranakan Chinese from a small number of samples from other Peranakan groups (supplementary table S1, Supplementary Material online) or with very recent admixture, we defined outliers as those >3 SD away from the mean in any of the top 3 PCs. We then iteratively removed the outliers and recalculated the means and SDs of PCs to identify additional outliers until convergence. We identified 15 outliers in total, including 9 self-reported Peranakan Chinese. Among the remaining 162 samples, 158 were self-reported Peranakan Chinese and 4 had no self-reported ancestry.

### ADMIXTURE Analysis

Similar to PCA, we applied ADMIXTURE (v1.3.0) on 944,059 autosomal SNPs and 113,037 chromosome X SNPs, respectively, under the unsupervised mode and the supervised mode with four reference populations (Alexander et al. 2009). For each *K* in the unsupervised analysis, 10 runs with different seeds were performed and the result with the highest likelihood was picked. We used the option –haploid=“male : 23” to accommodate the haploid genotypes for males in non-PARs of the X chromosome.

### RFMix Analysis

We inferred local ancestry using RFMix (v 1.5.4) (Maples et al. 2013) based on 983,282 bi-allelic autosomal SNPs and 155,049 bi-allelic SNPs on the X chromosome. We ran RFMix without expectation-maximization (EM) iterations, and with the PopPhased option and the minimal node size set to 5. Because RFMix required the number of haplotypes to be even in the reference panel, we discarded one male sample from each of the Indian, Malay, and European populations. For the study individuals, males were coded as homozygous diploid on the X chromosome. RFMix output posterior probabilities that each small window of a study haplotype came from one of the four reference ancestry populations. If none of the four posterior probabilities in a window was >0.9, the ancestry was set as unknown. We visualized the local ancestry of each individual using the karyogram plot colored by the inferred ancestry (Martin et al. 2017). From the inferred local ancestry, we calculated the global ancestry for each individual as the proportion of ancestry tract length (in unit of cM) contributed by each reference population, excluding tracts of unknown ancestry.

### *f_3_* Statistic

We calculated the *f*_3_ statistic using the program qp3Pop in AdmixTools 7.0.1 (Patterson et al. 2012) based on 953,064 bi-allelic autosomal SNPs with MAF > 0.05 and every pair of SNPs being at least 2 kb apart. Standard errors were estimated by the block jackknife method implemented in qp3Pop, and the blocks were automatically determined by qp3Pop (number of blocks = 727).

### Mitochondrial and Y Haplogroups

The MT and Y haplogroup analyses were restricted to Peranakan samples, SG Chinese, and three reference populations of Chinese, Malays, and Indians, whose raw sequencing data were available to us. We extracted reads mapped to MT or Y chromosome and the unmapped reads, and remapped these reads to the MT reference genome of the revised Cambridge Reference Sequence (rCRS) to infer the MT haplogroups using MToolBox (v 1.1) (Calabrese et al. 2014). To infer the Y haplogroups, we first called genotypes on the Y chromosome using bcftools and then inferred the Y haplogroups using callHaplogroups.py program of the yHaplo software (v 1.0.13) (Poznik 2016).

We developed the following approach to assess the maternal and paternal contribution from each reference population to the Peranakan Chinese. First, we computed the haplogroup frequencies in each reference population, denoted as $P\left(H|A\right)$, where *H* is an MT or Y haplogroup and *A* is an ancestral source (Chinese, Malay, or Indian). Given a haplogroup *H* observed in a Peranakan sample, we computed the posterior probability that *H* came from population *A* by the Bayes’ theorem: $P(A|H)=\frac{P(H|A)P(A)}{\sum_{A}P(H|A)P(A)}$, where the prior probability $P\left(A\right)$ was estimated from the global ancestry fractions inferred by RFMix analysis on autosomes. We estimated the maternal (for MT haplogroups) or paternal (for Y haplogroups) contribution from ancestry *A* as $\rho_{A}=\overset{-}{P}\left(A|H\right)$, in which the average was taken over all Peranakan samples. The ancestral source of an individual’s haplogroup can be modeled using a categorical distribution with event probabilities equaling P(A|H). To assess the distribution of $\rho_{A}$, we sampled with probability P(A|H) the ancestry for all individuals’ haplogroups for 1,000 realizations and calculated $\rho_{A}$ for each realization. The 95% CI was constructed by the 2.5 and 97.5 percentile from 1,000 realizations. The *P* value for the null hypothesis of $\rho_{A}=0$ was given by the fraction of realizations in which ancestry *A* was not sampled.

### GLOBETROTTER Analysis

We inferred the date and source compositions of admixture events using GLOBETROTTER (November 8, 2017) (Lawson et al. 2012; Hellenthal et al. 2014). We included Malays and all South and East Asian populations from 1KGP as the surrogate populations to infer admixture events in Peranakan Chinese and SG Chinese. All analyses were based on 983,282 bi-allelic autosomal SNPs. Following the manual of GLOBETROTTER, we used ChromoPainter (v2) to obtain the haplotype sample paintings and copying vectors. We then ran the GLOBETROTTER program to estimate admixture dates and source compositions by fitting the coancestry curves and assuming 29 years per generation, which were derived from the sample paintings and copying vectors. We set the grid range for coancestry curves to be 1–50 cM with bin size equal to 0.1 cM, in order to detect both ancient and recent admixture events. For analyzing SG Chinese, we also used a grid range of 1–10 cM with bin size equal to 0.01 cM. This setting allowed for more accurate inference of the ancient admixture event, because we found 99% of the exponential decay of coancestry curves occurred within 10 cM. We applied 100 bootstraps to evaluate the statistical significance of admixture events and the 95% CI of the estimated admixture dates.

## Supplementary Material

Supplementary data are available at *Molecular Biology and Evolution* online.

## Supplementary Material

msab187_Supplementary_Data
