# Supplementary material for: Genetic Admixture in the Culturally Unique Peranakan Chinese Population in Southeast Asia
Source: Mol Biol Evol. 2021 Jun 21;38(10):4463–74. doi: 10.1093/molbev/msab187 (PMC8476152; doi:10.1093/molbev/msab187)
Supplement: msab187_Supplementary_Data [file msab187_supplementary_data.zip › Peranakan_suppl 20210616.pdf]

**Supplementary Tables and Figures**

**Table S1. Demographic information of Peranakan samples.**

| Characteristic | Sample size or mean±sd |
|---|---|
| **Age** | 53.3±16.0 |
| **Self-reported sex** | |
| Male | 68 |
| Female | 107 |
| Not available | 2 |
| **Self-reported ancestry** | |
| Peranakan Chinese | 167 |
| Peranakan Caucasian | 1 |
| Peranakan Eurasian | 3 |
| Peranakan Indian | 1 |
| Not available | 5 |

**Table S2. Genetic relatedness among Peranakan samples.**

| Relatedness | Criteria | No. of pairs |
|---|---|---|
| Parent-offspring | $2^{-5/2}<\varphi<2^{-3/2}$ and $\pi_0<0.1$ | 23 |
| Full sibling | $2^{-5/2}<\varphi<2^{-3/2}$ and $\pi_0>0.1$ | 22 |
| 2nd degree | $2^{-7/2}<\varphi<2^{-5/2}$ | 18 |
| 3rd degree | $2^{-9/2}<\varphi<2^{-7/2}$ | 33 |
| Total | | 96 |

Notations: $\varphi$, kinship coefficient; $\pi_0$, the probability of zero identical-by-descent sharing.

**Table S3. Global ancestry fractions of different Chinese groups inferred by the supervised ADMIXTURE method.**

| Chinese group | Chr | Chinese % | Malay % | Indian % | European % |
|---|---|---|---|---|---|
| Peranakan | 1-22 | 93.9 (92.8-94.9) | 5.14 (4.16-6.12) | 0.76 (0.55-0.98) | 0.22 (0.13-0.31) |
| | X | 84.8 (80.4-89.2) | 12.7 (8.55-16.8) | 1.77 (0.90-2.63) | 0.74 (0.41-1.07) |
| Singapore | 1-22 | 97.7 (97.0-98.3) | 2.22 (1.54-2.90) | 0.07 (0.02-0.12) | 0.06 (0.03-0.09) |
| | X | 93.5 (91.3-95.6) | 5.64 (3.51-7.78) | 0.62 (0.25-1.00) | 0.26 (0.10-0.43) |
| Southern | 1-22 | 98.2 (97.7-98.8) | 1.50 (0.96-2.05) | 0.06 (0.02-0.09) | 0.21 (0.14-0.27) |
| | X | 96.1 (94.9-97.2) | 2.54 (1.51-3.57) | 1.02 (0.55-1.48) | 0.38 (0.17-0.59) |
| Northern | 1-22 | 98.1 (97.8-98.5) | 0.32 (0.00-0.64) | 0.37 (0.25-0.48) | 1.19 (1.00-1.38) |
| | X | 96.0 (94.6-97.3) | 1.95 (0.73-3.17) | 0.89 (0.52-1.26) | 1.21 (0.80-1.61) |

Mean and 95% CI (in parentheses) of ancestry fractions are presented. "Chr" stands for chromosome.

**Table S4. Testing admixture with Malays in four Chinese groups by the $f_3$ statistic.**

| Target | Source 1 | Source 2 | $f_3$ | $Z$ |
|---|---|---|---|---|
| Peranakan Chinese | Chinese | Malay | -0.000715 | -15.367 |
| Singapore Chinese | Chinese | Malay | -0.000143 | -2.756 |
| Southern Chinese | Chinese | Malay | 0.000586 | 8.222 |
| Northern Chinese | Chinese | Malay | 0.003058 | 33.264 |

**Table S5. Mitochondrial haplogroup distribution in Chinese, Malays, Indians, and Peranakan Chinese.**

| Macro-haplogroup | Chinese | | Malay | | Indian | | Peranakan Chinese | |
|---|---|---|---|---|---|---|---|---|
| | Counts | Percentage | Counts | Percentage | Counts | Percentage | Counts | Percentage |
| A | 30 | 3.0 | 2 | 0.5 | 0 | 0.0 | 0 | 0.0 |
| B | 185 | 18.6 | 90 | 22.6 | 0 | 0.0 | 21 | 18.3 |
| C | 25 | 2.5 | 0 | 0.0 | 0 | 0.0 | 3 | 2.6 |
| D | 160 | 16.1 | 6 | 1.5 | 1 | 0.2 | 9 | 7.8 |
| E | 5 | 0.5 | 50 | 12.5 | 0 | 0.0 | 12 | 10.4 |
| F | 193 | 19.4 | 68 | 17.0 | 3 | 0.5 | 22 | 19.1 |
| G | 26 | 2.6 | 1 | 0.3 | 2 | 0.3 | 0 | 0.0 |
| H | 0 | 0.0 | 0 | 0.0 | 28 | 4.5 | 0 | 0.0 |
| I | 0 | 0.0 | 0 | 0.0 | 2 | 0.3 | 0 | 0.0 |
| J | 1 | 0.1 | 0 | 0.0 | 7 | 1.1 | 0 | 0.0 |
| K | 0 | 0.0 | 0 | 0.0 | 1 | 0.2 | 1 | 0.9 |
| M | 270 | 27.1 | 125 | 31.3 | 373 | 59.3 | 35 | 30.4 |
| N | 47 | 4.7 | 23 | 5.8 | 12 | 1.9 | 5 | 4.3 |
| P | 0 | 0.0 | 1 | 0.3 | 1 | 0.2 | 0 | 0.0 |
| R | 34 | 3.4 | 26 | 6.5 | 91 | 14.5 | 5 | 4.3 |
| T | 0 | 0.0 | 0 | 0.0 | 7 | 1.1 | 0 | 0.0 |
| U | 0 | 0.0 | 0 | 0.0 | 90 | 14.3 | 0 | 0.0 |
| W | 0 | 0.0 | 0 | 0.0 | 9 | 1.4 | 0 | 0.0 |
| X | 0 | 0.0 | 0 | 0.0 | 1 | 0.2 | 0 | 0.0 |
| Y | 2 | 0.2 | 5 | 1.3 | 0 | 0.0 | 1 | 0.9 |
| Z | 18 | 1.8 | 2 | 0.5 | 1 | 0.2 | 1 | 0.9 |
| Total | 996 | 100 | 399 | 100 | 629 | 100 | 115 | 100 |

**Table S6. Y haplogroup distribution in Chinese, Malays, Indians, and Peranakan Chinese.**

| Macro-haplogroup | Chinese | | Malay | | Indian | | Peranakan Chinese | |
|---|---|---|---|---|---|---|---|---|
| | Counts | Percentage | Counts | Percentage | Counts | Percentage | Counts | Percentage |
| C | 14 | 4.3 | 11 | 7.3 | 6 | 2.7 | 3 | 6.7 |
| D | 6 | 1.8 | 0 | 0.0 | 0 | 0.0 | 0 | 0.0 |
| E | 0 | 0.0 | 0 | 0.0 | 1 | 0.4 | 0 | 0.0 |
| F | 0 | 0.0 | 2 | 1.3 | 0 | 0.0 | 0 | 0.0 |
| G | 0 | 0.0 | 1 | 0.7 | 5 | 2.2 | 0 | 0.0 |
| H | 0 | 0.0 | 3 | 2.0 | 75 | 33.6 | 0 | 0.0 |
| J | 0 | 0.0 | 4 | 2.6 | 31 | 13.9 | 0 | 0.0 |
| K | 0 | 0.0 | 2 | 1.3 | 0 | 0.0 | 0 | 0.0 |
| L | 0 | 0.0 | 0 | 0.0 | 48 | 21.5 | 0 | 0.0 |
| M | 0 | 0.0 | 1 | 0.7 | 0 | 0.0 | 0 | 0.0 |
| N | 15 | 4.6 | 0 | 0.0 | 0 | 0.0 | 2 | 4.4 |
| O1 | 95 | 29.0 | 93 | 61.6 | 5 | 2.2 | 9 | 20.0 |
| O2 | 194 | 59.1 | 23 | 15.2 | 0 | 0.0 | 31 | 68.9 |
| P | 0 | 0.0 | 1 | 0.7 | 0 | 0.0 | 0 | 0.0 |
| Q | 1 | 0.3 | 0 | 0.0 | 4 | 1.8 | 0 | 0.0 |
| R | 3 | 0.9 | 7 | 4.6 | 48 | 21.5 | 0 | 0.0 |
| S | 0 | 0.0 | 3 | 2.0 | 0 | 0.0 | 0 | 0.0 |
| Total | 328 | 100 | 151 | 100 | 223 | 100 | 45 | 100 |

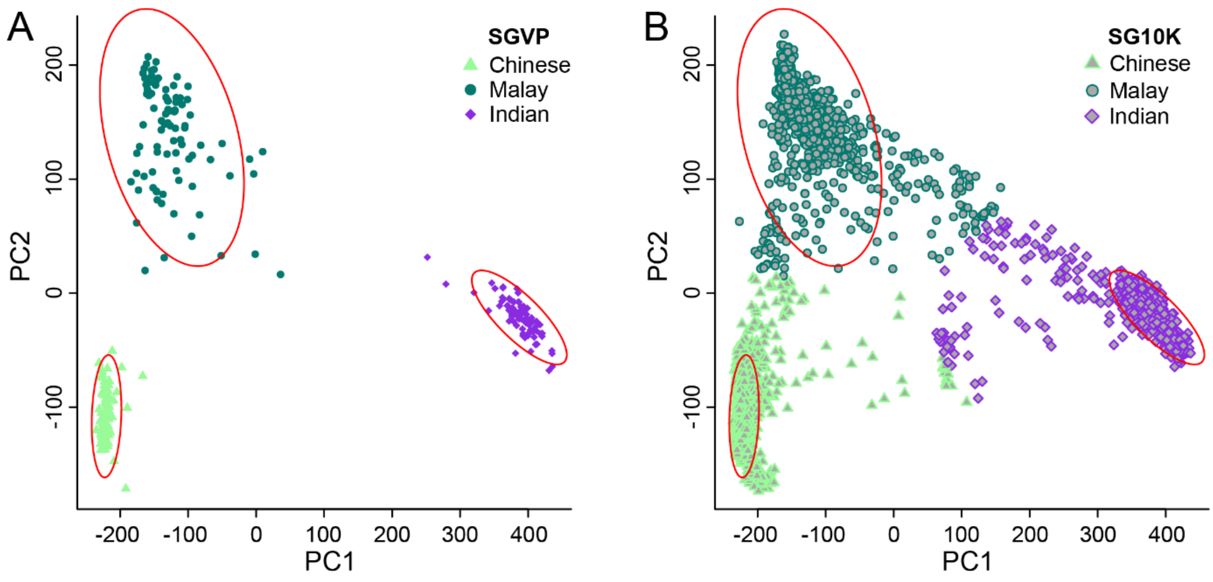


**Figure S1. Choice of reference individuals based on PC coordinates.** (A) PC coordinates of SGVP samples and the corresponding 95% concentration ellipses (solid red lines). (B) SG10K samples projected on the ancestry space created by SGVP samples (not shown) and SGVP confidence ellipses.

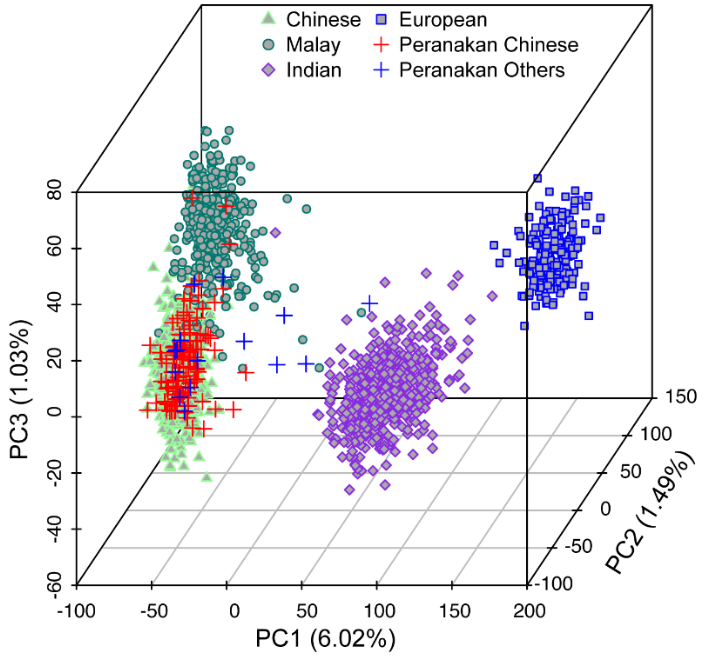


**Figure S2. Top 3 PCs of Peranakan samples based on 113,037 X SNPs.** Peranakan Chinese and Peranakan Others were defined based on autosomal SNPs as shown in **Figure 1**.

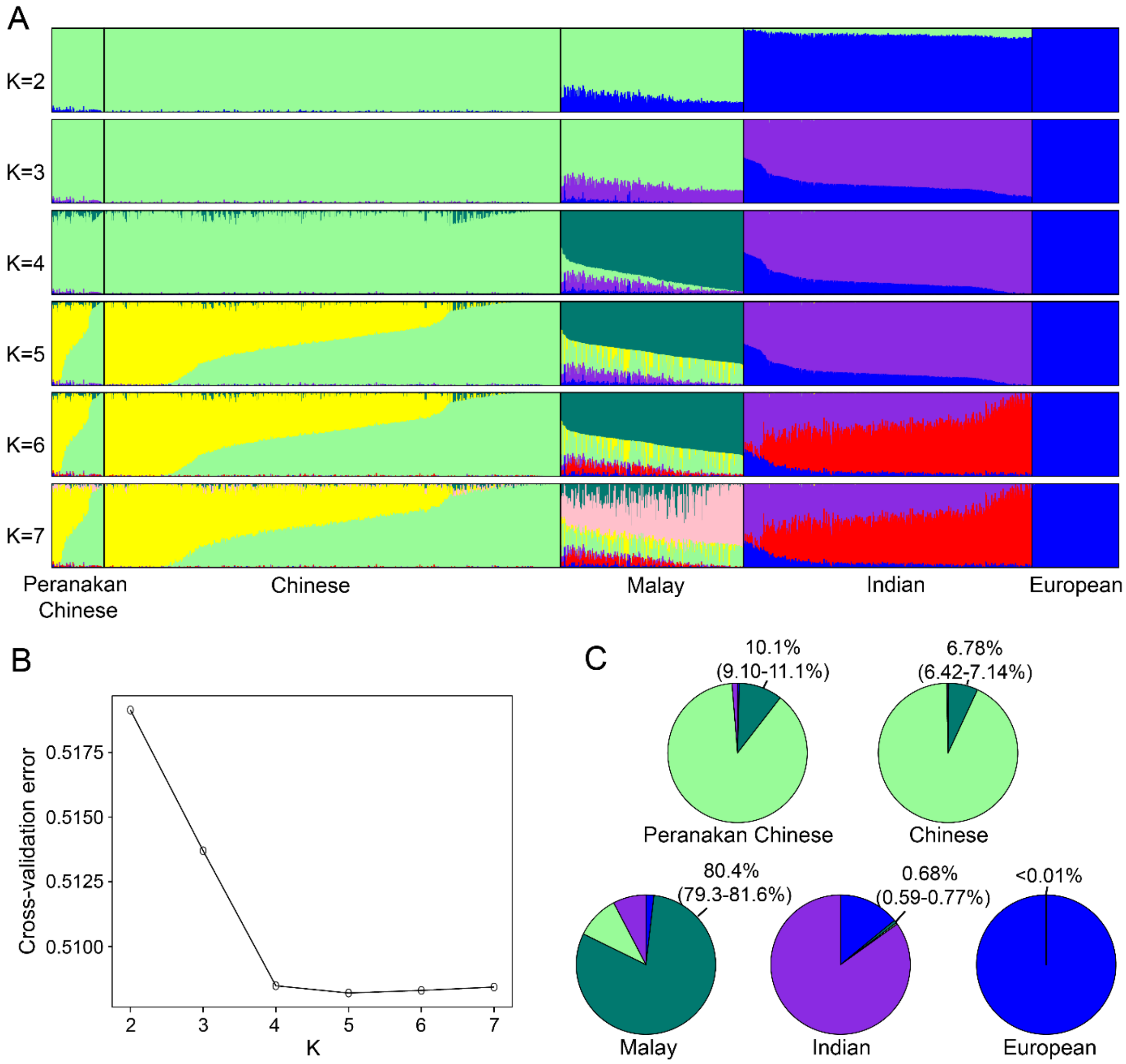


**Figure S3. Unsupervised ADMIXTURE analysis of Peranakan Chinese and four reference populations.** Unsupervised ADMIXTURE analyses were performed on biallelic autosomal SNPs with MAF>0.05 and at least 2 kb apart from each other. The number of hypothetical ancestral components, *K*, varied from 2 to 7. (A) Estimated ancestry fractions with different values of *K*. (B) Five-fold cross-validation errors for different *K*. While the minimum cross-validation error was achieved at *K*=5, there is only small difference compared to *K*=4, at which the ancestral components align well with the reference populations. (C) Pie charts of mean ancestry for each population when *K*=4. Mean and 95% CI of the Malay-like ancestral fraction (dark green) are indicated for each population.

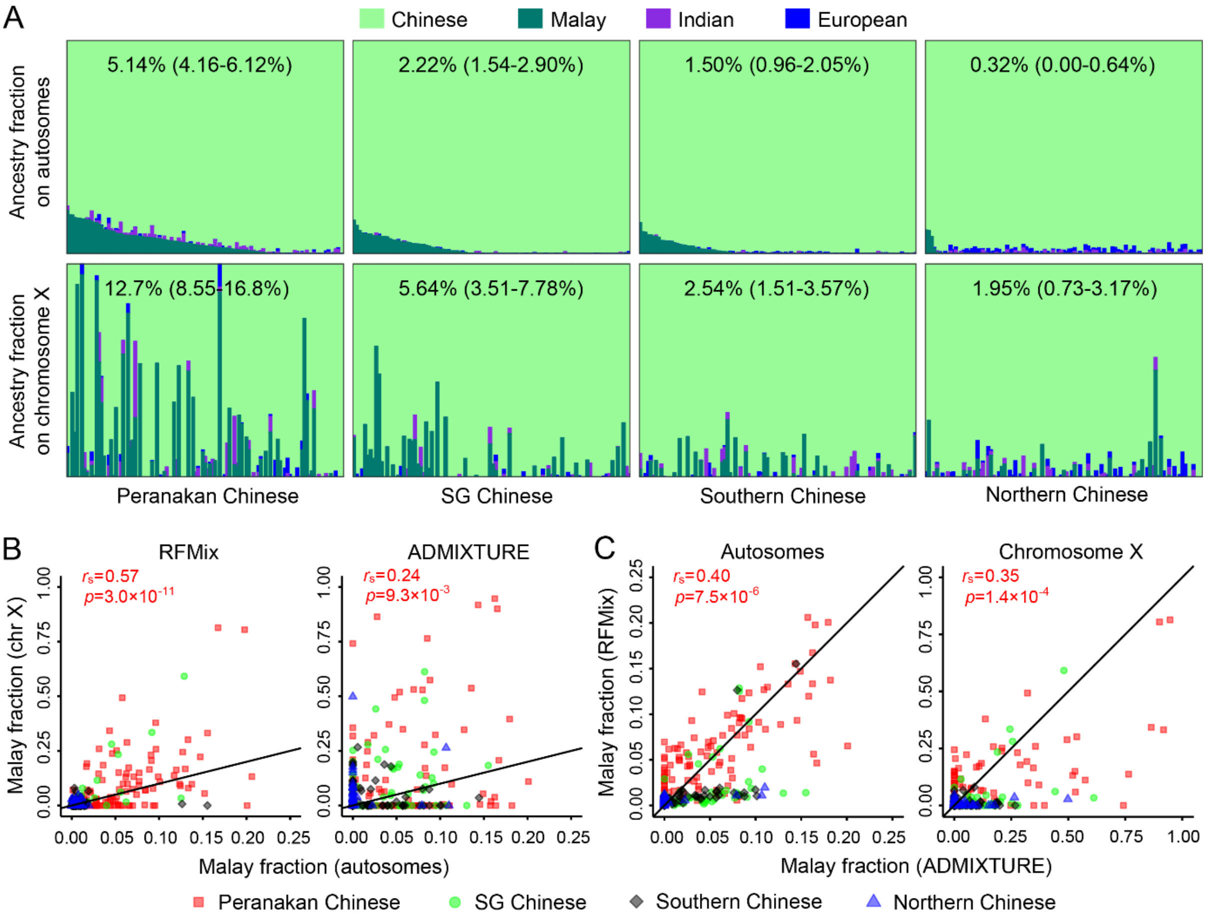


**Figure S4. Global ancestry fractions of Peranakan Chinese estimated by the supervised ADMIXTURE method.** (A) Ancestry fractions on autosomes and the X chromosome for Peranakan Chinese, SG Chinese, southern Chinese, and northern Chinese. Each bar represents the ancestry composition of one individual. Individuals on the top and bottom panels are in the same order. The mean and 95% CI of Malay ancestry for each population are labelled within each panel. (B) Comparison of the Malay ancestry fraction inferred using SNPs on chromosome X and autosomes by RFMix (left) or ADMIXTURE (right). Spearman's correlations and corresponding *p*-values for Peranakan Chinese are indicated in red text. (C) Comparison of the Malay ancestry fraction inferred by RFMix and ADMIXTURE using SNPs on autosomes (left) or chromosome X (right). Spearman's correlations and corresponding *p*-values for Peranakan Chinese are indicated in red text. The black line indicates the diagonal of y=x in panels B and C.

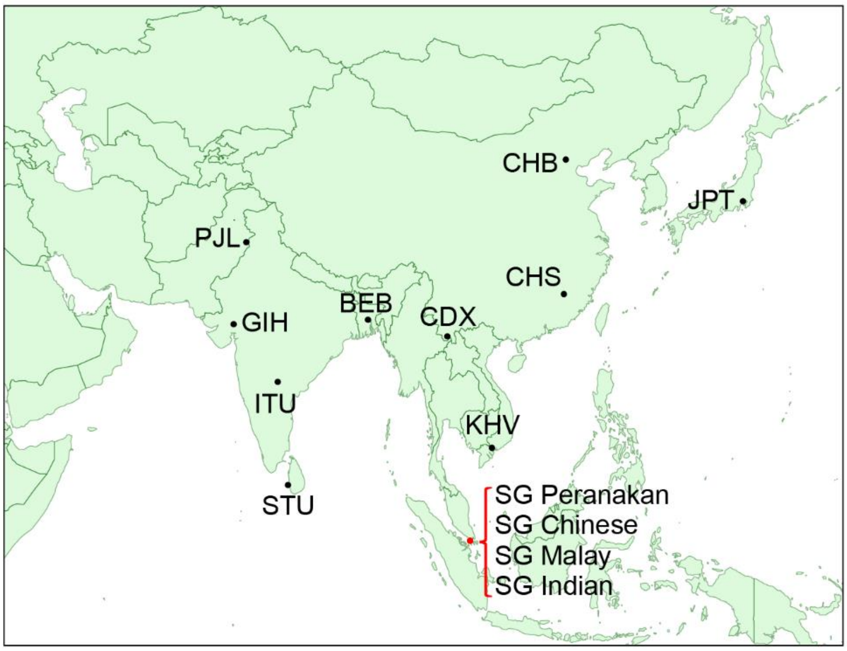


**Figure S5. Geographic distribution of Asian populations included in the analysis.** Abbreviations of 1KGP populations: BEB, Bengali; GIH, Gujarati; ITU, Telugu; PJL, Punjabi; STU, Sri Lankan Tamil; CDX, Chinese Dai; CHB, Han Chinese in Beijing; CHS, Southern Han Chinese; JPT, Japanese; KHV, Kinh in Vietnam.

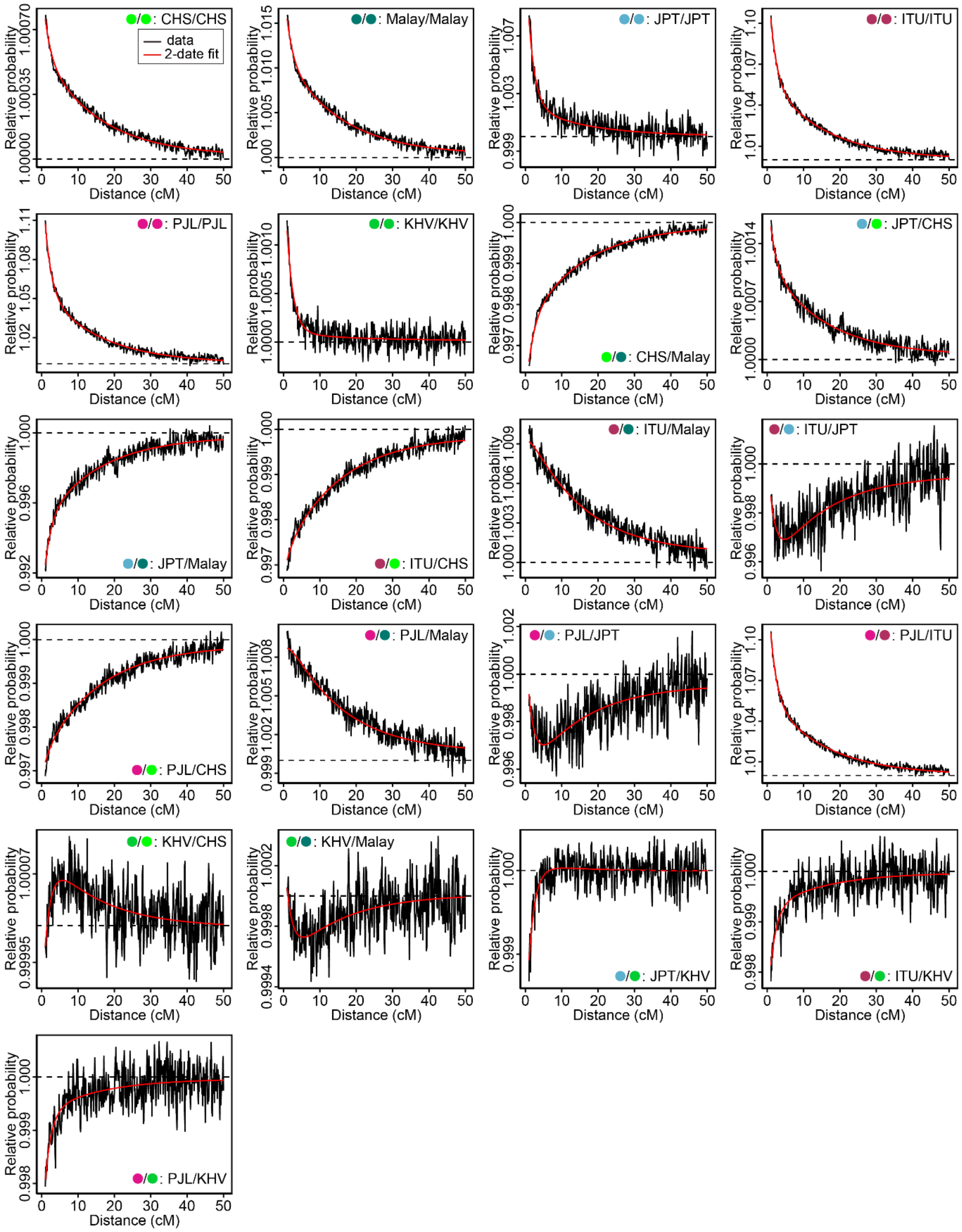


**Figure S6. Co-ancestry curves of selected pairs of populations in Peranakan Chinese.** Only populations with more than 2% contribution to an ancestral source in either of the two admixture events were included. The solid black curves were observed co-ancestry curves, and the solid red curves were 2-date fit estimated by GLOBETROTTER. A horizontal black dashed line at y=1.0 was drawn in every plot for visual aid.

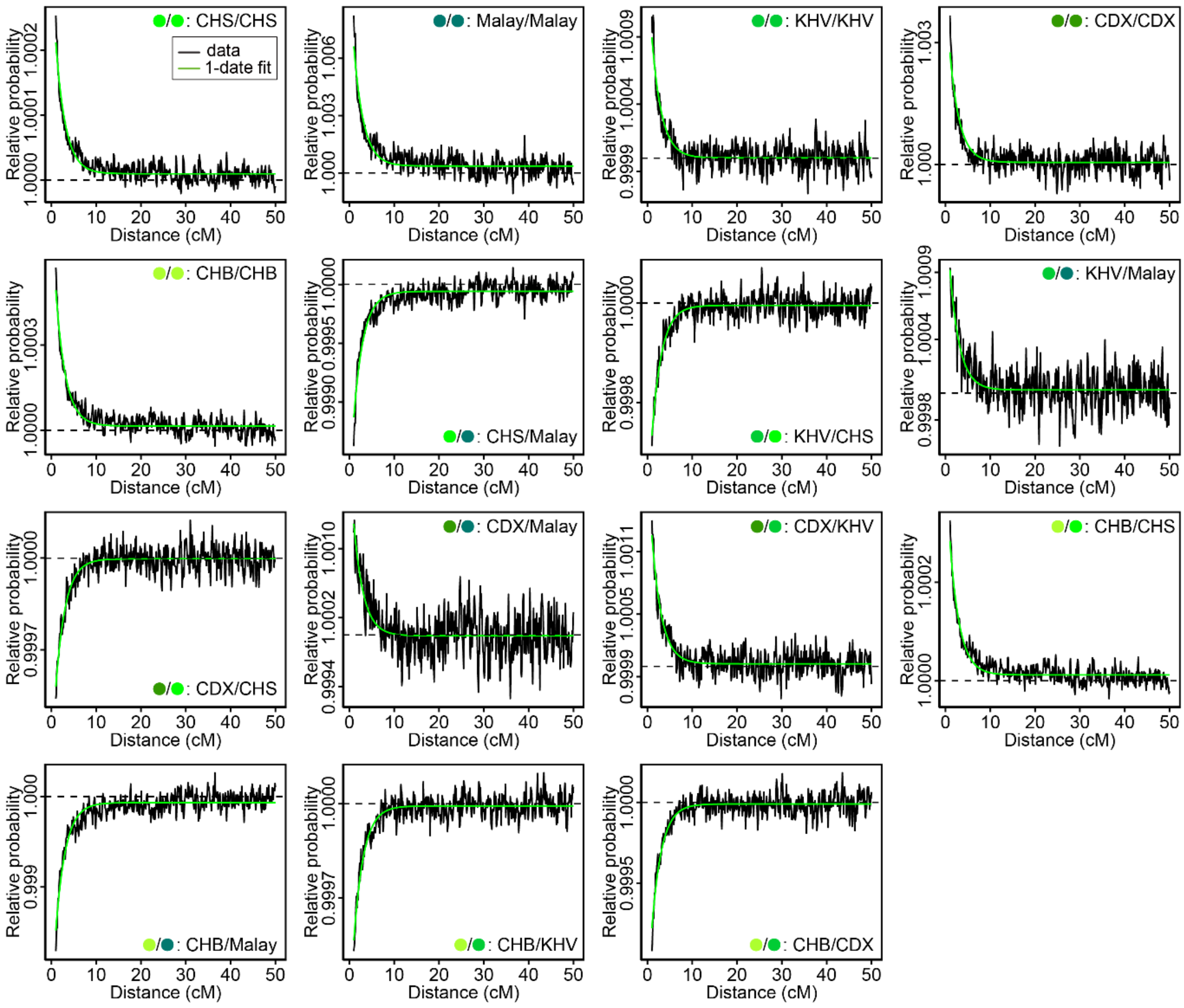

**Figure S7. Co-ancestry curves of selected pairs of populations in Singapore Chinese.** Only populations with more than 2% contribution to an ancestral source in the admixture event were included. The solid black curves were observed co-ancestry curves, and the solid green curves were 1-date fit estimated by GLOBETROTTER. A horizontal black dashed line at y=1.0 was drawn in every plot for visual aid.

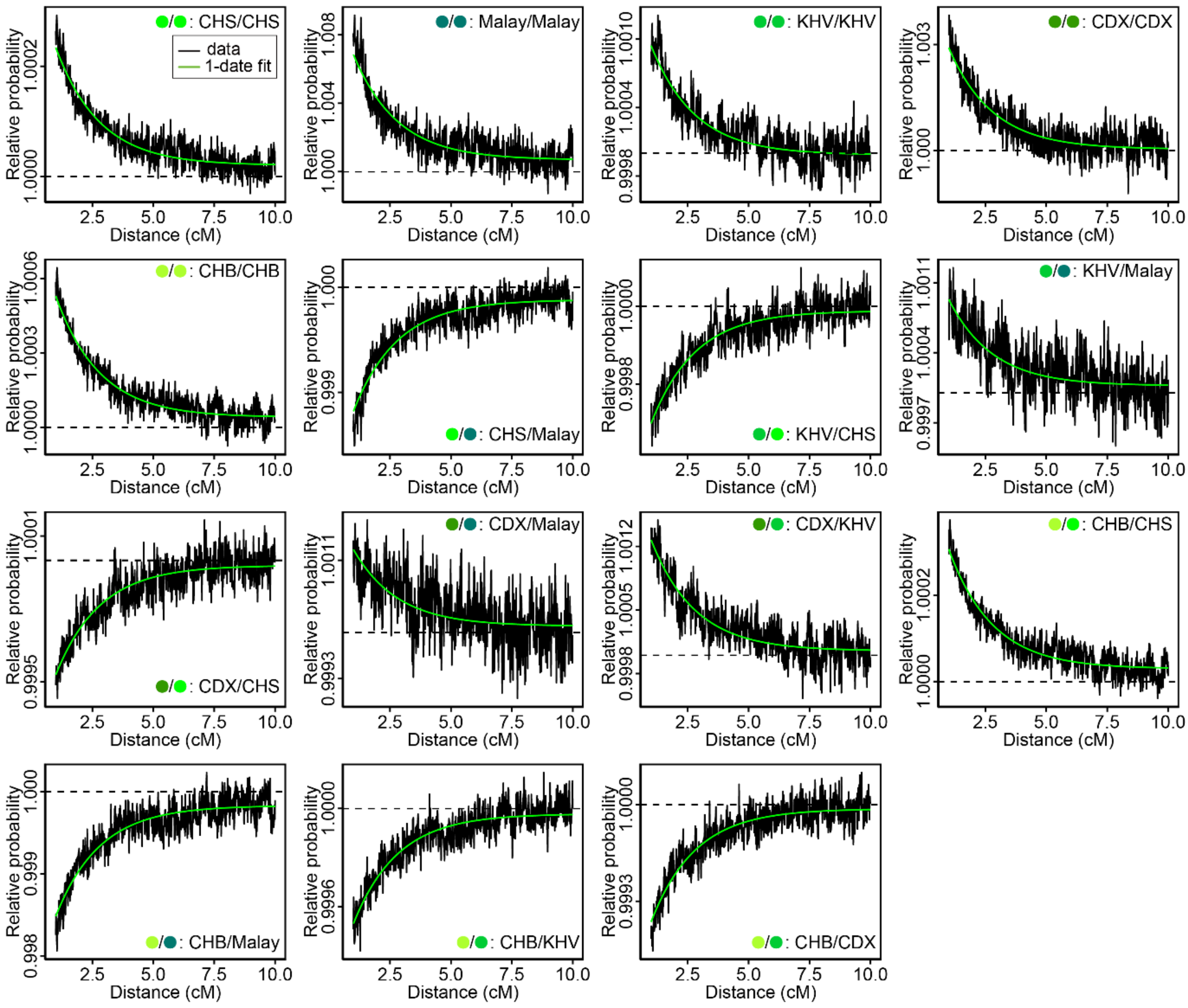


**Figure S8. Co-ancestry curves of selected pairs of populations in Singapore Chinese at a grid range of 1 to 10cM.** Only populations with more than 2% contribution to an ancestral source in the admixture event were included. The solid black curves were observed co-ancestry curves, and the solid green curves were 1-date fit estimated by GLOBETROTTER. A horizontal black dashed line at y=1.0 was drawn in every plot for visual aid.

**Supplementary Note**

**The SG Peranakan Project team (listed alphabetically)**

**Genome Institute of Singapore, A*STAR, Singapore**: Ruth Jinfen Chai, Jinzhuang Dou, Bangfen Pan, Zenia Tiang

**Huazhong University of Science and Technology, China**: Chaolong Wang, Degang Wu

**Indonesia International Institute for Life Sciences (i3L), Indonesia**: Amadeus Yeremia Pribowo, Ivanna Williantara, Rizky Nurdiansyah

**National University of Singapore, Singapore**: Edita Aliwarga, Chukwuemeka George Anene-Nzelu, Cheryl Xue Li Chan, Roger S. Y. Foo, Matthew Ackers Johnson, Jyn Ling Kuan, Peter Yiqing Li, Benson Tingsen Lim, Choon Kiat Lim, Shi Ling Ng, Wilson Lek Wen Tan, Ruifeng Wang
