# Supplementary material for: Genetic Admixture in the Culturally Unique Peranakan Chinese Population in Southeast Asia
Source: Mol Biol Evol. 2021 Jun 21;38(10):4463–74. doi: 10.1093/molbev/msab187 (PMC8476152; doi:10.1093/molbev/msab187)
Supplement: msab187_Supplementary_Data [file msab187_supplementary_data.zip › Response to reviewers' comments.docx]

**Point-by-point responses to the reviewers’ comments**

**Reviewer 1:**

In this study, the authors generated and analysed whole-genome sequencing data of 177 participants from Singapore Peranakan groups. The results suggest low levels (5-6%) of Malay admixture in the Peranakan Chinese population, primarily contributed by Malay females about two centuries ago. The analyses presented are reasonably compelling evidencing admixture and sex-bias admixture in the Peranakan Chinese population, supported by admixture patterns in uniparental markers and comparisons between autosomes and the X-chromosome. Further, the authors estimated the admixture dates to propose one hypothesis about the demographic history of East Asian and Southeast Asian groups. I have some concerns about the interpretation of the results in Figure 4, and the consistency of the proposed model (see comments below). Overall, this study extends previous knowledge about the admixture dynamics in the Peranakan Chinese population by generating high-quality data and using haplotype-based methods, thus I recommend publishing this manuscript after minor modifications.

**Response**: We thank the reviewer for appreciating the values of our work.

Major comments:
1) The interpretations of the co-ancestry curves in Figure 4A, 4D, and 4E are very **confusing**. They are not showing admixture between two Malay-related ancestries since the curves are going down in all the figures in the panel. I would expect to see the co-ancestry curves to go in the opposite direction (**going up**) to detect admixture between two sources. Thus, this figure needs to be better explained in the legend and main text, and the co-ancestry curves testing Malay/Southern Chinese (1/2 ancestries) admixture should be included in the figure or as supplementary figures.

**Response**: We apologize for the confusion. The GLOBETROTTER method actually fitted co-ancestry curves of all pairs of surrogate populations jointly. In each analysis, we included 11 surrogate populations (5 SAS, 5 EAS, and Malay), such that there were 66 co-ancestry curves in theory. We have now displayed co-ancestry curves between surrogate populations with >2% contribution to any ancestral source in **Figures S6-S8**. As the reviewer expected, we do see the Malay/CHS co-ancestry curve going up, confirming the admixture between a Malay-dominant and a Chinese dominant source. We have also rewritten the legend of Figure 4 (page 23) and the main text (page 8, lines 236-242) for clearer presentation.

2) It’s missing the ADMIXTURE plot including **ALL the populations that were used in the analysis that includes populations from the four reference panels**. It’s very important to know the **admixture pattern in the reference panel for each ancestry**. In case there is a reference panel with admixture (e.g. 20% Chinese-related ancestry in the Malay reference panel) the results could be different as we can see with the correlation between ADMIXTURE and RFMix results for autosomes in Suppl Figure S3C (different than in previous studies; e.g. Martin et al, AJHG 2017). To have “un-admixed” reference panels is very important since the RFMix analysis was performed without using the expectation-maximization (EM) algorithm.

**Response**: We thank the reviewer for the insightful comment. We agree with the reviewer that it is ideal for the reference populations to be free of admixture. That’s why we have selected reference individuals based on PCA plot, excluding samples that might be recently admixed (**Figure S1**).

Following the reviewer’s suggestion, we have now added unsupervised ADMIXTURE analysis to display population structure of Peranakan Chinese and the four reference populations (**Figure S3**; main text on page 6, lines 149-157; page 14, line 477 to page 15, line 480). But we note that in the unsupervised ADMXITURE analysis, shared ancestral components between populations could be attributed to demographic models other than genuine admixture (Lawson *et al.* 2018 *Nat Commun*). Specifically, in our study, southern Chinese and Malays might have shared ancestry related to Austronesian people, as inferred by our GLOBETROTTER analysis. Thus, we would prefer not to select reference samples based on unsupervised ADMIXTURE analysis.

In addition, we would like to point out that the weak correlation between ADMIXTURE and RFMix results is not surprising when the admixture fraction is small, as in our case for the Malay component. For example, in the reference provided by the reviewer (Martin et al. 2017, *AJHG*), the correlation between RFMix and ADMIXTURE drop substantially for the Native American component, which was present at low levels in the ACB population (see Figure S4A in Martin *et al.* 2017, *AJHG*). Such observations were also well documented in Tables S5-S6 of Fortes-Lima *et al.* (2017, *AJHG*). Furthermore, the small genetic distance between Chinese and Malay (*F*_ST_=0.012) increases the uncertainty in estimating Malay admixture fraction (we have discussed this limitation on page 12, lines 359-362), and thus contributes to the low correlation between ADMIXTURE and RFMix estimates.

References:

- Lawson DJ, van Dorp L, Falush D. A tutorial on how not to over-interpret STRUCTURE and ADMIXTURE bar plots. *Nat Commun*. 2018; 9(1):3258.
- Martin AR, Gignoux CR, Walters RK, Wojcik GL, Neale BM, Gravel S, Daly MJ, Bustamante CD, Kenny EE. Human Demographic History Impacts Genetic Risk Prediction across Diverse Populations. *Am J Hum Genet*. 2017;100(4):635-649.
- Fortes-Lima C, Gessain A, Ruiz-Linares A, Bortolini MC, Migot-Nabias F, Bellis G, Moreno-Mayar JV, Restrepo BN, Rojas W, Avendaño-Tamayo E, Bedoya G, Orlando L, Salas A, Helgason A, Gilbert MTP, Sikora M, Schroeder H, Dugoujon JM. Genome-wide Ancestry and Demographic History of African-Descendant Maroon Communities from French Guiana and Suriname. *Am J Hum Genet*. 2017 Nov 2;101(5):725-736.

3) GLOBETROTTER analysis is performed for each population independently, thus results for several populations should be interpreted with caution when they are put together like in Figure 5, particularly when putative sources are missing in the analysis (such as early Austronesian or early Malay). Thus, the manuscript would strongly benefit from additional analyses, I would recommend testing the proposed admixture history in Figure 5 using model-testing approaches such as qpGraph or qpAdm, where the authors can indeed test admixture models like the one proposed in the study.

**Response**: We appreciate the reviewer’s suggestion. We understand the reviewer’s concern that Figure 5 is a speculated model based on separated GLOBETROTTER analyses rather than a directly inferred model. That’s why we present Figure 5 in the Discussion section rather than in the Results.

Following the reviewer’s suggestion, we applied qpGraph to test the admixture model, based on genotype data of Peranakan Chinese, Chinese, Malays and Europeans. Based on **Figure 5**, we proposed the model in **Figure R1**, which incorporates genetic drift from the source populations to the present-day Chinese and Malays. The analysis was based on 953,064 bi-allelic autosomal SNPs (MAF>0.05 and at least 2kb apart). Due to computational limitation and to avoid impact of unbalanced population sample sizes, we down-sampled Chinese and Malays to 100 samples each, and repeated qpGraph analysis 20 times. Mean and SD of the estimated branch lengths and admixture fractions from 20 runs were displayed in **Figure R1**. The max Z score is less than 0.53 in all 20 runs, suggesting high quality of model fit. The inferred genetic drift between M and Malay suggest the source Malay population contributing to the Peranakan Chinese might be different from the present-day SG Malays, which could be partly due to sampling issue given the genetic diversity in Southeast Asia.

Overall, the qpGraph results support our proposed admixture model in **Figure 5**, except for some minor quantitative differences in the estimated admixture fractions. We believe the GLOBETROTTER results are more informative by including many surrogate populations and modelling the LD patterns, in comparison to the frequency-based qpGraph method. We would also like to point out that although we applied GLOBETROTTER analysis on each target population separately, we have minimized the discrepancy between analyses, by using the same parameter settings and surrogate populations, to facilitate interpretation and comparison of results. For these reasons and considering that our **Figure 5** is presented as an illustrative diagram in the Discussion section, we prefer to not to add the new qpGraph results to avoid complications.

**Figure R1. Admixture graph with branch lengths and admixture fractions inferred from qpGraph.** Each node represents a sampled population (shaded in light blue) or a hypothetical population (shaded in white). A solid arrow indicates genetic drift, measured by the *f*_2_ statistic (×1,000) besides. A dashed arrow indicates genetic contribution to an admixed population, indicated by the blue text besides. All estimates were presented as mean±SD from 20 runs of qpGraph. The max Z score was close to 0 (mean and maximum across 20 runs were 0.13 and 0.53, respectively), suggesting excellent model fit.

Minor comments:
4) I recommend changing expressions like “two-date-two-party model” or “multiple-date-two-party model”, for more clear terms like “two-way admixture model with two-dates” or “two-way admixture model with multiple-dates”.

**Response**: We thank the reviewer for the suggestion. In the context of GLOBETROTTER analysis, an admixture model is characterized by the number of admixture pulses and the number of source populations. So we prefer to change “multiple-date-two-party model” to “multiple-date-two-way admixture model” throughout the manuscript. Furthermore, we added explanation of “multiple-date-two-way admixture model” in the main text for clearer presentation (page 8, lines 245-246).

5) Page 6: There is a typo, the word “that” is twice in this sentence: “, the average fraction of Malay ancestry on the X chromosome is higher *than that* on the autosomes”.

**Response**: Thanks. The typo has been fixed.

6) Page 7, last paragraph: To clearly distinguish between two-dates and multiple-dates admixture models, I recommend changing “one pulse” for “two pulses” in the sentence: “a multiple-date-two-party model, which involves more than *one pulse* of admixture between two source populations.”

**Response**: Thank you for the suggestion. The original GLOBETROTTER paper (Hellenthal *et al.* 2014, *Science*) referred “multiple-date admixture” to the scenario where “a null hypothesis of admixture at a single time in the past” was rejected (see page 32 in the supplementary materials of the paper). Following this definition, “multiple-date/pulse” includes “two-date/pulse”. Thus, we prefer to keep “one pulse” to avoid the risk of generating confusion with the literature.

7) Page 10: In the Discussion about the difficulties to detect post-admixture selection signals, it’s worth to mention that it’s notably low (5-6%) the contribution from the Malay population to the Peranakan Chinese population. The three signals described in the Results section have ~10% Malay-related ancestry in the present-day Peranakan Chinese population, which it’s indeed a really low value for selected genetic regions.

**Response**: We agree with the reviewer that given the challenge to detect post-admixture selection signals in Peranakans, our results in this section are weak. The same issue was also raised by reviewer 2. Thus, we decide to remove the post-admixture selection analysis from the manuscript and focus on the demographic history of Peranakans.

8) Page 11, last sentence in Conclusions: I would recommend changing “our human race” for “our species”  or “human populations” in the sentence: “Our study, together with many other examples, demonstrates the power of genetic analysis in complementing historical evidence in revealing the past of *our human race*.”

**Response**: Thanks. We have replaced our concluding sentence with a more specific statement to reiterate the significance of the study (page 12, lines 371-373), so this comment no longer applies.

9) Page 16, GLOBETROTTER paragraph: There is a typo, the word “for” is twice in this sentence: “This setting allowed *for for* more accurate inference of the ancient admixture event,”

**Response**: Thanks. We have corrected the typo.

10) Supplementary figures: A map for the locations of the samples from this study and comparative populations would be useful for international readers.

**Response**: Thank you for the suggestion. We added a map in **Figure S5**.

**Reviewer 2:**
This paper studied the genetic history of the Peranakan Chinese in Singapore. The dissection of the genetic structure of the Peranakan Chinese was carried out. In accordance with the written history, the analysis showed a sex bias admixture suggesting a major contribution of females to the Peranakan Chinese genome, again in line with the common wisdom of the history of the Malay land. In particular, the contact between the Chinese and the Malay land started at the beginning of the century; There has been a Chih Tu kingdom in the Malay land 1,500 years ago; During the Ming Dynasty, the Malacca Sultan married a princess from China called Hang Li Po, which brought along her attendants to Malacca. That formed among the earliest wave of immigration of Chinese to Malay Land. I have a few concerns which I hope the authors can address in a revised version.

**Response**: We thank the reviewer for the careful evaluation of our work and historical input. We are aware of the legend about Princess Hang Li Po. While there is a widespread theory that the princess and her attendants were founders of the Peranakan Chinese community, this theory is highly controversial and has little evidence to support.

1) Dating of admixture. When the Malacca kingdom was down by the Portuguese in 1511, the descendant of the Sultan ran to Singapore and formed the Johorian kingdom in the 1600s. That was when Singapore started to develop. There may have been some Peranakan Chinese since then inhabited in Singapore. That says the dating of the Peranakan may be more recent than that anticipated. Of course, the massive immigration happened ~200 years ago during British colonization. I would suggest if the authors could estimate the admixture time using mtDNA and Y-Chr, if this could be done, I suspect the picture could be clearer.

**Response**: We thank the reviewer for the suggestion, but we think it is infeasible to date the admixture event using mtDNA and Y chromosome for two reasons. First, there is no recombination in mtDNA and Y chromosome due to their uniparental inheritance, so we are not able to date the admixture based on the distribution of local ancestry tracts. Second, both mtDNA and Y chromosome have small genome sizes so that there would be very few mutations to accumulate within a few generations since admixture.

2) Another concern of mine is, Malay themselves are an admixed population. I would expect that this admixture could affect the estimated gene flow to Peranakan Chinese.

**Response**: If the admixture in Malays occurred before the admixture with Chinese in forming the Peranakan community, our admixture inference for Peranakan Chinese should not be affected. What to be concerned is the recent admixture in Malays that lead to substantial difference between our sampled Malays and the source Malays admixed with Chinese. To mitigate such impact, we have purposely selected our Malay reference individuals based on the PCA results (**Figure S1**) to avoid recent admixed samples. In our previous study (Wu *et al.* 2019, *Cell*), we have studied the same set of Malay samples, and inferred that the best-guess admixture model for Malay is a one-date-two-way model with the estimated date at ~1,700 years ago, way before the formation of Peranakan Chinese. While we cannot completely rule out the existence of recent admixture in our Malay samples, we believe this issue should have introduced little bias in the admixture inference for Peranakans.

Reference:

- Wu D, Dou J, Chai X, Bellis C, Wilm A, Shih CC, Soon WWJ, Bertin N, Lin CB, Khor CC, DeGiorgio M, Cheng S, Bao L, Karnani N, Hwang WYK, Davila S, Tan P, Shabbir A, Moh A, Tan EK, Foo JN, Goh LL, Leong KP, Foo RSY, Lam CSP, Richards AM, Cheng CY, Aung T, Wong TY, Ng HH; SG10K Consortium, Liu J, Wang C. Large-Scale Whole-Genome Sequencing of Three Diverse Asian Populations in Singapore. Cell. 2019; 179(3):736-749.

3) Please clarify in the **main text** whether the 177 Singapore (SG) Peranakans are a part of the SG10K and whether they were included in the previous publication.

**Response**: We have clarified in the main text that these samples were sequenced as part of the SG10K Project and 79 out of 177 samples have previously been included in the SG10K Pilot study (page 13, lines 381-382).

4) How did the authors evaluate the influence on their analysis of potential batch effects resulted from the combination of different data sets from different sources?

**Response**: We thank the reviewer for raising this issue. We are confident that our results are not subjected to batch effects for several reasons. First, as part of the SG10K project, we have used exactly the same experimental protocol and bioinformatics pipeline to process our Peranakan samples and the reference Chinese, Malays, and Indians. In particular, all the sequenced samples were jointly called using a LD-based algorithm, which have been shown to achieve excellent genotyping accuracy even for low-coverage samples (see Figure 1 in Wu *et al.* 2019, *Cell*). Second, in our analyses that involved data from the 1KGP, we have focused on common SNPs with MAF>0.05 and were present in both 1KGP and SG10K call sets, excluding multi-allelic SNPs, INDELs, SNPs within 5 bp of any INDELs, and SNPs with mismatched alleles (see the SNP QC section on page 14); such stringent QC procedure ensures high-quality genotypes in both datasets and thus minimizes the batch effects. Third, we have used the same approach to jointly analyze SG10K and 1KGP samples in our SG10K Pilot study (Wu *et al.* 2019, *Cell*), and we did not observe noticeable batch effects in our extensive PCA and ADMIXTURE results (see Figure 3, Figure S4, and Figure S6 in Wu *et al.* 2019, *Cell*).

5) The authors observed a weak correlation between RFMix and Admixture results and chose to report that based on RFMix. I would suggest a different method be applied so that the inconsistency might be better explained.

**Response**: Please see our response to the second major comment of Reviewer 1, in which we explained that the weak correlation between ADMIXTURE and RFMix results is not surprising when the admixture fraction is small. Same observations have been reported by Martin *et al.* (2017, *AJHG*) and Fortes-Lima *et al.* (2017, *AJHG*). We have followed Fortes-Lima *et al.* (2017, *AJHG*) to report results from RFMix in the main text and those from ADMIXTURE in the supplementary materials. In fact, it has been shown by simulation studies that the haplotype-based RFMix method outperforms the frequency-based ADMXITURE method in determining the ancestry fractions in complex admixed populations (Uren, Hoal, and Möller 2020, *BMC Genet*). We have now added this justification to the main text (page 7, lines 194-198).

Reference:

- Uren C, Hoal EG, Möller M. Putting RFMix and ADMIXTURE to the test in a complex admixed population. *BMC Genet*. 2020; 21(1):40.

6) I am not convinced by the selection analysis, although it was only a small portion. It does not gel up nicely with the overall story, and the signal does not provide any important information. More importantly, the authors should justify they were “post-” admixture selection.

**Response**: We agree with the reviewer that the selection signals are relatively weak, and obscure the main messages of the paper. This issue was also raised by reviewer 1. Therefore, we have decided to remove the selection analysis from this manuscript and focus on demographic history.

7) A more general comment from me is, the significance of the study would be reiterated clearer. I think it is too general by emphasising the power of genetic analysis, which is well known.

**Response**: We thank the reviewer for this constructive thought. The significance of our study is specifically on understanding the origin of Peranakan Chinese and revealing the dispersal history of Chinese and their interaction with indigenous populations in Southeast Asia, as we previously stated in the abstract. Following the reviewer’s suggestion, we have replaced our concluding sentence with a more specific statement to reiterate the significance of the study (page 12, lines 371-373).

8) I am not so sure about any advantage of WGS data over genotyping data in studying the Peranakans history, at least I do not see that from this paper. Can the authors make it clear?

**Response**: We have focused on scientific findings in the paper without emphasizing the advantages of WGS data over genotyping data. There are two key advantages. First, WGS data are free of potential Eurocentric ascertainment bias (Patterson *et al.* 2012, *Genetics*), which is particularly important for genetic studies of Asian populations. Second, WGS data provide complete genetic information, including mtDNA and Y-chromosome, which are generally not well captured in genotyping data (depending on the chip design), but are very informative in our inference of sex-biased admixture history. We have now highlighted these two points in the Discussion section (page 9, lines 270-272 and line 280).

Reference:

- Patterson N, Moorjani P, Luo Y, Mallick S, Rohland N, Zhan Y, Genschoreck T, Webster T, Reich D. Ancient admixture in human history. *Genetics*. 2012; 192(3):1065-93.

9) I would interpret the “Malay ancestry” in southern Chinese (0.86%) and that in northern Chinese (0.25%) to be artificial results. In this sense, the proportion of “Malay ancestry” in Peranakan Chinese and that in SG Chinese are likely overestimated, or underestimated. The authors are suggested to make a more reliable estimation using some other methods.

**Response**: We have extensively reviewed the literature and RFMix is still the state-of-the-art method for inferring admixture fractions. In our comparison, both unsupervised and supervised ADMIXTURE analyses estimated even higher Malay fractions in southern and northern Chinese.

While we do not find a better method to estimate admixture fraction, we have made some additional analysis and text editing to help interpretation of the results. First, we have performed the *f*_3_ tests, results from which suggested admixture with Malays in Peranakan Chinese and SG Chinese, but not in southern and northern Chinese (page 6, lines 160-164; page 16, lines 497-501; Table S4). Second, we have added explanation that “the low levels of Malay ancestry estimated in southern and northern Chinese might reflect shared common ancestry rather than genuine admixture events” (page 6, lines 174-176). In particular, Malays are expected to have a higher level of shared ancestry with southern Chinese than with northern Chinese due to ancient admixture events with Austronesian people. Overall, the Malay fractions in southern and northern Chinese are one order of magnitude lower than that in the Peranakan Chinese, and should not compromise our conclusions.

10) Introduction, “The Peranakan Chinese are descendants of early Chinese traders from the southern provinces of China”, is there any literature supporting this statement?

**Response**: Yes, we have now added two references to support this statement (Song 1984; Chia 2015). While we are aware of the legend about Princess Hang Li Po, whether the princess and her attendants were founders of Peranakan Chinese is highly controversial and has little concrete evidence. The theory that the Peranakan Chinese are descendants of early Chinese traders is more widely accepted. We also rewritten the sentence as “It is widely believed that the Peranakan Chinese are descendants of early Chinese traders from the southern provinces of China (Song 1984; Chia 2015).” Please see page 3 lines 67-69 in the Introduction.

References:

- Chia, Felix. 2015. The Babas (The Estate of Felix Chia Thian Hoe, Landmark Books Pte Ltd: Singapore).
- Song, Ong Siang. 1984. One Hundred Years' History of the Chinese in Singapore (Oxford University Press: Singapore ; New York).
